# Structure and electron transfer pathways of an electron-bifurcating NiFe-hydrogenase

**DOI:** 10.1126/sciadv.abm7546

**Published:** 2022-02-25

**Authors:** Xiang Feng, Gerrit J. Schut, Dominik K. Haja, Michael W. W. Adams, Huilin Li

**Affiliations:** 1 Department of Structural Biology, Van Andel Institute, Grand Rapids, MI, USA.; 2 Department of Biochemistry and Molecular Biology, University of Georgia, Athens, GA, USA.

## Abstract

Electron bifurcation enables thermodynamically unfavorable biochemical reactions. Four groups of bifurcating flavoenzyme are known and three use FAD to bifurcate. FeFe-HydABC hydrogenase represents the fourth group, but its bifurcation site is unknown. We report cryo-EM structures of the related NiFe-HydABCSL hydrogenase that reversibly oxidizes H_2_ and couples endergonic reduction of ferredoxin with exergonic reduction of NAD. FMN surrounded by a unique arrangement of iron sulfur clusters forms the bifurcating center. NAD binds to FMN in HydB, and electrons from H_2_ via HydA to a HydB [4Fe-4S] cluster enable the FMN to reduce NAD. Low-potential electron transfer from FMN to the HydC [2Fe-2S] cluster and subsequent reduction of a uniquely penta-coordinated HydB [2Fe-2S] cluster require conformational changes, leading to ferredoxin binding and reduction by a [4Fe-4S] cluster in HydB. This work clarifies the electron transfer pathways for a large group of hydrogenases underlying many essential functions in anaerobic microorganisms.

## Teaser

NiFe-hydrogenase structures reveal FMN, three Fe-S clusters, and protein conformation changes involved in electron bifurcation.

## INTRODUCTION

Flavin-based electron bifurcation is a recently recognized mechanism of energy coupling found in many microorganisms (*1*). Bifurcating oxidoreductase-type enzymes can catalyze the endergonic reduction of low-potential, high-energy substrates by simultaneously coupling them to the reduction of high-potential, low-energy substrates in an exergonic reaction. For example, bifurcating reactions enable NADH (reduced form of nicotinamide adenine dinucleotide) [*E*′ –280 mV, under physiological conditions; (*1*)] to be used as the source of electrons to reduce the low-potential redox protein ferredoxin [Fd; *E*′ ~−500 mV, under physiological conditions; (*1*)], which, in turn, is a critical electron donor in many fundamental metabolic processes, including nitrogen fixation and the production of methane and hydrogen gas. Electron bifurcation is therefore used by microorganisms as a general mechanism to produce low-potential, high-energy electrons without the expenditure of adenosine triphosphate (ATP).

Four groups of phylogenetically unrelated flavin-based electron-bifurcating (BF) enzymes have evolved independently, and three of them have been structurally characterized [reviewed in (*1*, *2*)]. The first group is the electron transfer flavoproteins (ETFs) that have an EtfAB core that couples the oxidation of NADH and reduction of Fd to the reduction of high-potential substrates such as ubiquinone (*E*°′ +70 mV) or enoyl–coenzyme A (CoA)/acyl-CoA derivatives (*E*°′ ~0 mV). The core is composed of two lobes connected by a flexible linker. The large lobe includes EtfA and the EtfB N terminus and binds the electron-bifurcating flavin adenine dinucleotide (FAD), and the small lobe is the C-terminal domain (CTD) and binds the electron-transferring FAD. Structural studies have shown how the Fd-reducing core couples with other functional units, such as butyryl-CoA dehydrogenase (*3*, *4*), caffeyl-CoA reductase (*5*), and menaquinone oxidoreductase (*6*, *7*). The second group of bifurcating enzymes is exemplified by the NADH-dependent Fd NADP oxidoreductase (Nfn) transhydrogenase complex that contains an NfnAB core that couples the simultaneous oxidation of NADH and reduced Fd to drive the reduction of NADP for biosynthesis (*8*). The third group is represented by the MvhADG-HdrABC complex that contains hydrogenase (Mvh) and heterodisulfide reductase (Hdr) activities and uses H_2_ gas (*E*°′ −414 mV) to simultaneously reduce a heterodisulfide (*E*_m_ −140 mV) and Fd (*9*, *10*). In all three groups of structurally characterized BF-enzymes, the site of electron bifurcation is an unusual flavin designated BF-FAD. This splits electron pairs and diverts each to high- and low-potential pathways, where the low-potential electron transfer is driven by a highly reactive flavin intermediate [*E*_m_ ~−900 mV in Nfn; (*8*)].

The fourth group of BF-enzymes is represented by the HydABC FeFe-hydrogenases (*11*–*13*). The HydA subunit is highly homologous to the structurally characterized non-BF monomeric FeFe-hydrogenase (*14*) that contains a novel six-iron atom H-cluster at the catalytic site as well as three [4Fe-4S] and one or two [2Fe-2S] clusters arranged in a Y-shape. In the trimeric BF-FeFe-hydrogenases, HydB is predicted to contain one flavin and three [4Fe-4S] clusters and one [2Fe-2S] cluster, while HydC contains a single [2Fe-2S] cluster. The monomeric non-BF FeFe-hydrogenases oxidize Fd and evolve H_2_ gas (*14*), but the trimeric BF-FeFe-enzyme can only catalyze this reaction if NADH is also present (Eq. 1) (*12*). The reverse of electron bifurcation, or confurcation, therefore allows NADH (*E*′ −280 mV) and Fd (*E*′ ~−500 mV) to provide electrons for proton reduction (*E*°′ −414 mV).

$$\text{NADH}+2{\text{Fd}}_{\text{red}}+3H^{+}⇌\text{NAD}+2{\text{Fd}}_{\text{ox}}+2H_{2}$$ (1)

**Fig. 1.**
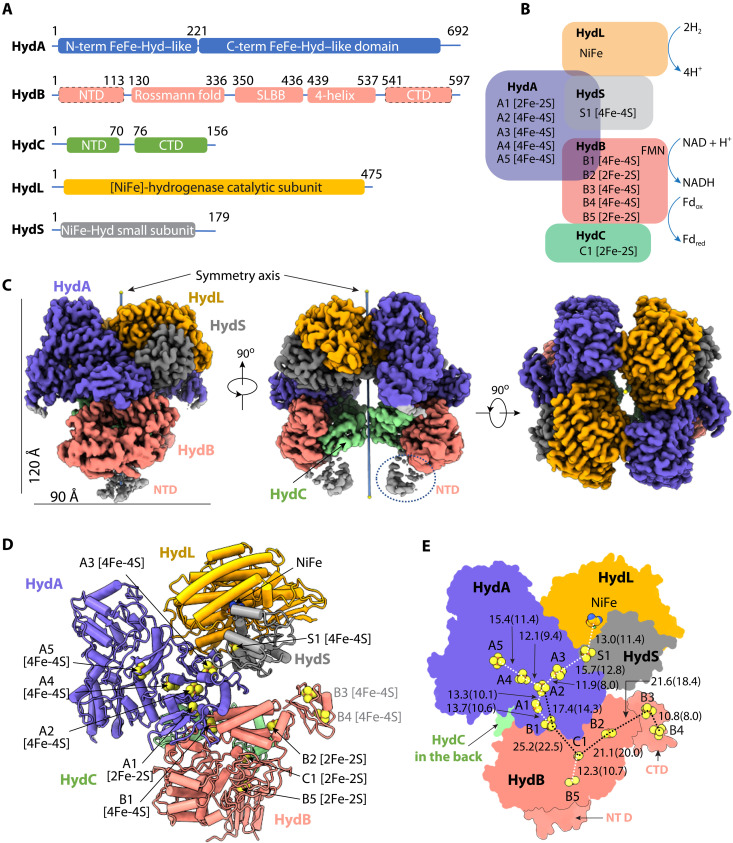
Structure of the *A. mobile* NiFe-HydABCSL decamer in the flavin-free apo state. (**A**) Domain structure of the five subunits. The NiFe-HydB NTD and CTDs are partially flexible, indicated by dashed outlines. (**B**) Subunit organization of the NiFe-Hyd complex and their associated cofactors. (**C**) Cryo-EM 3D map of the twofold symmetric NiFe-Hyd decamer in three orthogonal views. Subunits are individually colored. The vertical line marks the twofold axis. At this display threshold, the HydB Fd-like NTD is only partially visible, and the Fd-like CTD is invisible. (**D**) Atomic model in cartoon with cofactors in spheres. (**E**) Arrangement of the cofactors with distances labeled. The distances are measured in Å from center to center, with edge-to-edge values in parentheses.

The electron-bifurcating [FeFe]-HydABC hydrogenase (FeFe-HydABC) was first discovered in the anaerobic thermophile *Thermotoga maritima* (*12*), and several similar enzymes have now been characterized from other anaerobic bacteria (*15*–*19*). However, it is unclear what the nature is of the electron bifurcation center in this fourth group of BF-enzymes. Three sites of bifurcation have been proposed, namely, a second flavin (*13*, *20*), the catalytic FeFe-H-cluster (*2*, *21*), and unique arrangements of iron sulfur clusters (*22*, *23*). Here, we have purified another type of BF-hydrogenase, from the anaerobic bacterium *Acetomicrobium mobile*, that contains homologs of the three FeFe-ABC subunits but lacks the H-cluster and contains two additional subunits that harbor a NiFe-based catalytic site. NiFe-hydrogenases have been extensively characterized (*24*), and the prototypical enzyme consists of a heterodimer where the large (HoxH) subunit contains the NiFe catalytic site. Electrons are shuttled to it by a small (HoxY) subunit that contains three [4Fe-4S] clusters (*24*). However, the *A. mobile* enzyme is heteropentameric in which homologs of these small (S) and large (L) subunits are present, in addition to homologs of FeFe-HydABC. The motif that coordinates the catalytic H-cluster is missing in NiFe-HydA. We will refer to this new type of BF-hydrogenase as the NiFe-HydABCSL enzyme, where the HydABC module is equivalent to that in the FeFe-enzyme, and this module is predicted to contain 11 FeS clusters (*25*). The LS module is equivalent to a conventional NiFe-hydrogenase with the L subunit coordinating the NiFe-cluster, although there is only one cluster, rather than the expected three [4Fe-4S] clusters, in the S subunit (Fig. 1, A and B).

Here, we report cryo–electron microscopy (cryo-EM) structures of NiFe-HydABCSL determined in a flavin-free apo state, in an “electron bifurcation-ready” (BR) state, and in a “post-electron bifurcation” (PB) state. The HydABC module of the NiFe-HydABCSL holoenzyme is highly homologous to FeFe-HydABC (fig. S1) except that its HydA does not contain the residues that coordinate the catalytic H-cluster in FeFe-HydA. The structures of the NiFe-enzyme show that none of the previous proposals for the site of bifurcation in [FeFe]-HydABC hydrogenases are likely to be correct and that instead electron bifurcation occurs at a single NAD(H)-binding flavin in combination with a unique arrangement of three iron-sulfur clusters. Capturing the enzyme in multiple states has allowed us to describe the structural dynamics associated with electron transfer events.

## RESULTS AND DISCUSSION

### The *A. mobile* NiFe-HydABCSL hydrogenase assembles into a dimer-of-pentamers

A NiFe-HydABCSL enzyme has not been previously reported. *A. mobile* is an anaerobic bacterium growing optimally near 60°C that was isolated from a wastewater treatment plant (*26*). We noticed in its genome (*27*) an apparent operon that contained homologs of bifurcating FeFe-HydABC, although the HydA subunit appeared to lack the catalytic H cluster, adjacent to two genes encoding a heterodimeric (SL) NiFe-type hydrogenase (fig. S2A). The presence of a bifurcating NiFe-hydrogenase would fit with the metabolism of *A. mobile* as it grows by fermenting peptides and carbohydrates and produces H_2_ (*26*). We found that fructose-grown *A. mobile* produced close to a 2:1 ratio of H_2_ and acetate with no other reduced fermentation products (fig. S2B), similar to the situation with glucose-grown *T. maritima*, which contains a bifurcating FeFe-hydrogenase (*12*). We therefore purified the enzyme responsible for the hydrogenase activity in the cytoplasmic extract of *A. mobile* to homogeneity by multistep chromatography under anaerobic conditions (fig. S2, C and D). The purified enzyme contained five subunits by SDS gel analysis (fig. S2E) that, by mass spectrometry (MS) analysis, corresponded to five genes (Anamo_1675–1678 and 1681) in the operon corresponding to NiFe-HydABCSL. Three additional genes present in the operon, designated HydD, HydP, and HisK, are assumed to be involved in maturation and/or regulation of the NiFe-enzyme.

The estimated mass of the NiFe-HydABCSL enzyme according to size exclusion chromatography is ~500 kDa, while the calculated total mass is 233 kDa, suggesting that the enzyme assembles into a dimer of heteropentamers (fig. S2F). Inductively coupled plasma (ICP)–MS analysis of the pure enzyme showed that it contained approximately 32 atoms of Fe and 0.7 atoms of Ni per molecule of enzyme (HydABCSL), with a Fe:Ni ratio of 42.1 ± 0.5, which is in excellent agreement with the calculated value of 41:1, based on eight [4Fe-4S] clusters, four [2Fe-2S] clusters (which, as discussed below, includes one cluster not previously predicted by bioinformatic analyses), and a NiFe site per heteropentamer. The enzyme lost flavin during purification as the purified enzyme contained only approximately 0.35 flavin per mole, and this was identified as flavin mononucleotide (FMN) by MS analysis (fig. S2G). Native *T. maritima* FeFe-HydABC hydrogenase lost all of its FMN during purification using multiple chromatography steps, and a *K*_d_ (dissociation constant) of 0.9 μM was reported for the purified enzyme (*12*).

To facilitate both the structural and functional studies of NiFe-HydABCSL, we also purified Fd from *A. mobile* (fig. S3A). This protein (encoded by Anamo_0968) is highly similar (52% identity, 75% similarity) to the Fd of *T. maritima* (Tm_0927), which contains a single [4Fe-4S] cluster [*E*_m_ = −453 mV; (*28*)]. Using the *A. mobile* Fd, we found that under an atmosphere of H_2_, NiFe-HydABCSL reduced the Fd only if NAD was also added (fig. S3B) and both Fd and NAD were reduced simultaneously (fig. S3C). This H_2_-dependent bifurcation activity was stimulated by the addition of FMN and by FAD by approximately 35 and 10%, respectively. In comparison, *T. maritima* FeFe-HydABC was about 50% active when FAD was added in place of FMN (*12*). Both the cytoplasmic extract and the purified enzyme also evolved H_2_ in the physiological confurcation reaction in the presence of NADH and reduced Fd but only if both were present (fig. S3, D and E). Therefore, NiFe-HydABCSL catalyzes a reversible and tightly coupled bifurcation reaction, similar to what was reported for the FeFe-HydABC enzyme (*12*).

### The NiFe-HydABCSL structure reveals the pathways of electron transfer

**Fig. 2.**
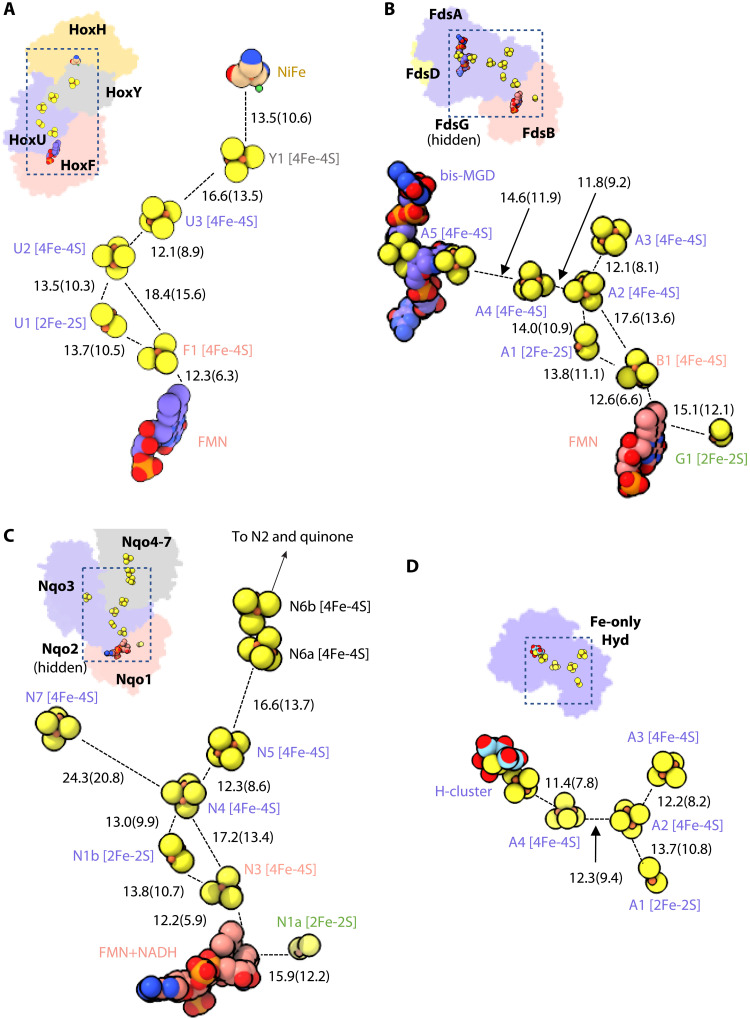
The electron transfer pathway in four homologs of NiFe-HydABCSL. Three nonbifurcating hydrogenase complexes sharing homologous subunits with NiFe-HydABCSL are aligned and shown in the same orientation for comparison: the NiFe-hydrogenase HoxFHUY (PDB ID 5XF9) (**A**), the formate dehydrogenase (FDH; PDB ID 6TGA) (**B**), the hydrophilic domain of respiratory complex I (PDB ID 3IAM) (**C**), and the single-subunit FeFe-hydrogenase (PDB ID 1FEH) (**D**). Inserted at the top left corner of each panel is the overall structure, with subunits individually colored. Equivalent subunits are in the same color. The distances are measured in Å from center-to-center, with edge-to-edge values in parentheses.

Great care was taken during the EM grid preparation procedure to minimize exposure of the NiFe-HydABCSL sample to air (see below). Several cryo-EM class averages had a clear mirror symmetry (fig. S4), indicative of the presence of a twofold symmetry in the particles, confirming the dimer-of-pentamers architecture predicted by the gel filtration analysis. After three-dimensional (3D) classification and refinement of the cryo-EM images, we obtained a twofold symmetric 3D map at an average resolution of 3.2 Å in a flavin-free apo state (Fig. 1C, figs. S5 to S7, and table S1). The final atomic model contained most sequences, all 12 predicted FeS clusters, and the catalytic NiFe site (Fig. 1, C and D, and fig. S8). The overall architecture of NiFe-HydABCSL resembles several nonbifurcating complexes, including *Hydrogenophilus thermoluteolus* HoxFHUY hydrogenase (*29*), the NADH-dependent formate dehydrogenase (FDH) from *Rhodobacter capsulatus* (*30*), and the NADH quinone oxidoreductase (complex I) of *Thermus thermophilus* (Fig. 2) (*31*). NiFe-HydB and NiFe-HydC of the BF-enzyme are fused and equivalent to HoxF of the non-BF hydrogenase and share a sequence identity of 32 and 23%, respectively. HoxU is a truncated version of NiFe-HydA and is 36% identical to the N-terminal equivalent of NiFe-HydA. The NiFe-HydA structure was fully resolved and is highly similar to the non-BF FeFe-HydA hydrogenase (*14*), with an N-terminal domain (NTD) coordinating the A1 [2Fe-2S] cluster and the A2, A3, and A4 [4Fe-4S] clusters and a CTD containing the A5 [2Fe-2S] cluster (fig. S8). However, the motif that binds the catalytic H-cluster in the CTD of the monomeric non-BF FeFe-hydrogenase (*14*) was not evident, and it was replaced by the A5 [4Fe-4S] cluster. NiFe-HydA is also homologous to FdsA (19% identity) of the *R. capsulatus* FDH FdsABDG complex (*30*), as well as to Nqo3 (20% identity) of the *T. thermophilus* NADH quinone oxidoreductase (complex I) (Fig. 2) (*31*). NiFe-HydC has an NTD and a CTD where the Fd-like CTD coordinates the C1 [2Fe-2S] cluster and is homologous to those in FdsG (34% identity) and Nqo2 (25% identity). But the iron-sulfur cluster is absent in HoxF of the non-BF hydrogenase. In addition to these nonbifurcating complexes, the NiFe-HydABC subunits are homologous to the bifurcating FeFe-HydABC hydrogenase from *T. maritima*, with sequence identities of 19, 54, and 42%, respectively (fig. S1).

NiFe-HydB has five well-resolved domains (Fig. 1A and fig. S8). The three middle domains (residues 113 to 536) include a Rossmann fold containing the FMN/NADH-binding site, a soluble ligand binding β-grasp (SLBB) domain, and a small four-helix bundle that coordinates the B1 [4Fe-4S] and B2 [2Fe-2S] clusters. While the B1 location can be predicted by conservation, the B2 [2Fe-2S] binding site was not previously thought to bind an FeS cluster in the bifurcating FeFe-hydrogenase or in related enzymes (*25*). However, in bifurcating FeFe-HydABC, an additional [2Fe-2S] not predicted by bioinformatics analysis was found in the separated HydB subunit by a combination of metal analysis and electron paramagnetic resonance spectroscopy (*32*). In a recent x-ray structure of a homologous domain, the anomalous Fe signal that was detected also suggests the presence of an iron-sulfur cluster (*33*). No density for the expected FMN was observed in the EM map, indicating that the as-purified complex is in the flavin-free apo state. The NTD and CTD of NiFe-HydB were partially flexible, although EM densities were clearly visible. The NTD is predicted to have a thioredoxin-like fold and binds one [2Fe-2S] cluster, while the CTD is predicted to have an Fd-like fold and to coordinate two [4Fe-4S] clusters. We used the online server Robetta (*34*) to derive homolog-based models for both domains and docked them in the EM densities. The structure reveals that the NiFe-HydB CTD coordinates B3 and B4 [4Fe-4S] clusters, while the NTD stabilizes the CTD of the NiFe-HydC and contains the B5 [2Fe-2S] cluster (fig. S9). The combination of clusters B2 to B5 is unique to bifurcating hydrogenases and absent in the nonbifurcating homologous Hox, FDH, and complex I enzymes. However, overall, the NiFe-HydB structure resembles the corresponding subunits HoxF, FdsB, and Nqo1 in these nonbifurcating enzymes, indicating a common ancestry for all these complexes.

The NiFe-HydSL structure is similar to the heterodimeric HoxHY of non-BF NiFe-hydrogenase (*24*), with a highly conserved catalytic module in NiFe-HydL. NiFe-HydL and HydS have 36 and 47% identity with their non-BF counterparts in the *H. thermoluteolus* HoxFHUY (*29*). NiFe-hydrogenases are O_2_ sensitive. While the NiFe site is coordinated by four cysteine residues in the reduced state, a proximal glutamate participates in coordination when the NiFe site is oxidized by O_2_ exposure (*29*). In our NiFe-HydSL structure, the NiFe site is coordinated by four cysteines only and the conserved proximal glutamate does not participate in the [NiFe] coordination (fig. S10). Therefore, NiFe-HydSL is in the reduced active state and brief air exposure during EM grid preparation did not oxidize the catalytic center. Flavoenzymes typically have a lower affinity for flavin in their reduced states (*29*, *35*), and in this regard, the absence of FMN in the NiFe-HydB structure is consistent with the reduced state of HydSL in our structure.

The NiFe-HydABCSL enzyme resembles a bivalved clam (Fig. 1C). Each “valve” consists of four subunits, HydA, HydB, HydL, and HydS, and is 90 Å wide and 120 Å tall. The remaining two HydC subunits are inside the clamshell and contact each other with a small interface of 183 Å^2^. HydB and HydL are at the hinge joint connecting the two valves with a large interface of 1742 Å^2^. However, the cofactors are beyond electron transfer distance between the two pentamers (>40 Å), indicating that each HydABCSL is a functional unit, and that dimerization may simply stabilize the structure. Within each HydABCSL pentamer, the largest subunit HydA is wedged between HydB and HydC from below and the NiFe-module HydSL from above (Fig. 1D). Because of this overall architecture, the H_2_-activating/evolving [NiFe] site is positioned at the very top of an electron transfer branch with only one possible path for electrons starting at the S1 [4Fe-4S] cluster, and thus, the NiFe site is unable to fulfill a bifurcation function (Fig. 1E). Moreover, our structure also disapproves the previous proposal (*21*) that the H-cluster of bifurcating FeFe-hydrogenases may function as the electron bifurcation center, as this would be located at the equivalent of the A5 [4Fe-4S] cluster in NiFe-HydABCSL enzyme, which is also at the terminus rather than at the branchpoint of an electron transfer pathway (Fig. 1, D and E).

### The “Y-shaped” FeS clusters of NiFe-HydABCSL are unlikely to be the bifurcation site

NiFe-HydABCSL contains a total of 12 FeS clusters. Two of them (A2 and C1) are in the centers of two “Y” shapes of FeS clusters and thus potential branch points for electron transfer (Fig. 1E). The Y of FeS clusters in FeFe-HydABC, with A2 linking A1, A3, and A4, has been proposed as the bifurcation site (*22*), although with our discovery of the fifth cluster in NiFe-HydB, we would predict a similar second Y of clusters, with C1 linking B1, B2, and B5, in the FeFe-hydrogenases. The Y clusters are a conserved feature among the homologous bifurcating complexes and to varying degrees in homologous nonbifurcating complexes and are the center of different electron transport pathways (*25*). They serve as the core to connect peripheral electron transfer branches that has allowed for the evolution of different functions (see Fig. 2, A to D). The FMN binding site is at the bottom of the clam (determined by cryo-EM as discussed below). The NiFe-HydA Y clusters connect FMN at the bottom with the hydrogenase catalytic NiFe at the top to form a linear chain of FMN-B1-A1-A2-A3-S1-[NiFe] (Fig. 1, D and E). Note that this path does not include clusters A4 and A5, which form the third arm of Y. The distances between the A5-A4-A2 clusters are within electron transfer range, but since A5 is at the dead end, this branch is most likely nonfunctional. Consistent with this, the A4/A5 branch is in the region that forms the dimer interface of HydABCSL, apparently stabilizing the quaternary organization.

The “Y-shape” cluster arrangement is not unique to the BF-enzymes, as the corresponding clusters in the non-BF hydrogenase Hox complex are linked similarly in a chain of FMN-F1-U1-U2-U3-Y1-[NiFe] (Fig. 2A), while missing the A4/A5 branch. In FDH, a similar Y-shape of FeS cluster links FMN to the catalytic molybdopterin (MGD) site via a chain of FMN-B1-A1-A2-A4-A5-MGD using the A4/A5 branch (Fig. 2B). In complex I, the Y-shape links FMN to the quinone binding site via a chain of FMN-N3-N1b-N4-N5-N6a-N6b-N2-quinone (Fig. 2C). A simpler Y-shape is present in monomeric nonbifurcating FeFe-hydrogenase where A5 is replaced by the H-cluster (Fig. 2D). We also predicted based on homology that this A4/A5 branch is used in the BF-FeFe-HydABC hydrogenases as well. Therefore, the Y cluster appears to have been maintained throughout evolution where electron transfer between any of the three arms is possible depending on the enzyme’s function. This means that the previous proposal that the Y-shape cluster arrangement is the site of bifurcation in the FeFe-HydABC enzymes is not correct (*22*). A similar conclusion was reached by a bioinformatic analysis of all enzymes that contain the Y cluster (*25*). Hence, in NiFe-HydABCSL, neither the catalytic NiFe site nor the Y cluster is a viable site for electron bifurcation.

### The branch with C1 and B2 [2Fe-2S] clusters is the low-potential electron pathway

The potential electron transfer branch C1-B2-B3-B4 is unique to the bifurcating NiFe- and FeFe-hydrogenase complexes. None of these four FeS clusters are observed in the non-BF NiFe-hydrogenase. The equivalent of the C1 [2Fe-2S] cluster is found in FDH (G1) and complex I (N1a), but they likely play a role in the accumulation of oxygen-sensitive species rather than thermodynamic coupling (see Fig. 2, B and C) (*36*). C1 sits at the center of the Y cluster linking B1, B2, and B5, but B5 is at a dead end (like A5) and is unlikely to be involved in electron transfer. However, B5 is close enough to the C1 cluster (11 Å) for electron transfer in the FMN-free apo state, so the possibility of B5 being involved in electron transfer to Fd cannot be entirely ruled out. Direct electron transfer between B1 and C1 is highly unlikely based on our structure, as the closest (side-to-side) distance between them is more than 22 Å, which is too far for electron transfer to occur. This large distance between B1 and C1 is also true in homologous proteins like complex I [19.4 Å; Protein Data Bank (PDB) 3IAM] and FDH (19.5 Å, PDB 6TGA). It therefore appears that the C1-B2-B3/B4 pathway transfers electron to Fd in the NiFe-enzyme since Fd is not an electron acceptor for the homologous non-BF complexes. It is not obvious where Fd should bind. In the monomeric non-BF FeFe-hydrogenase, A3 is the proposed binding site for Fd (*14*), but this site is blocked by the dimer interface in NiFe-HydABCSL. In Fd-dependent complex I type-I NAD(P)H dehydrogenase-like (NDH-I), the Fd binding site is formed by both NdhI and NdhH (*37*). NdhH is a homolog of NiFe-HydL, but access to NiFe-HydL is blocked by NiFe-HydA. However, NdhI shares an Fd fold and aligns well with the CTD of NiFe-HydB, suggesting that this CTD may bind Fd similar to the manner in which NdhI binds Fd. This makes B3 and B4 the prime candidates for low-potential electron transfer to Fd (fig. S11).

**Fig. 3.**
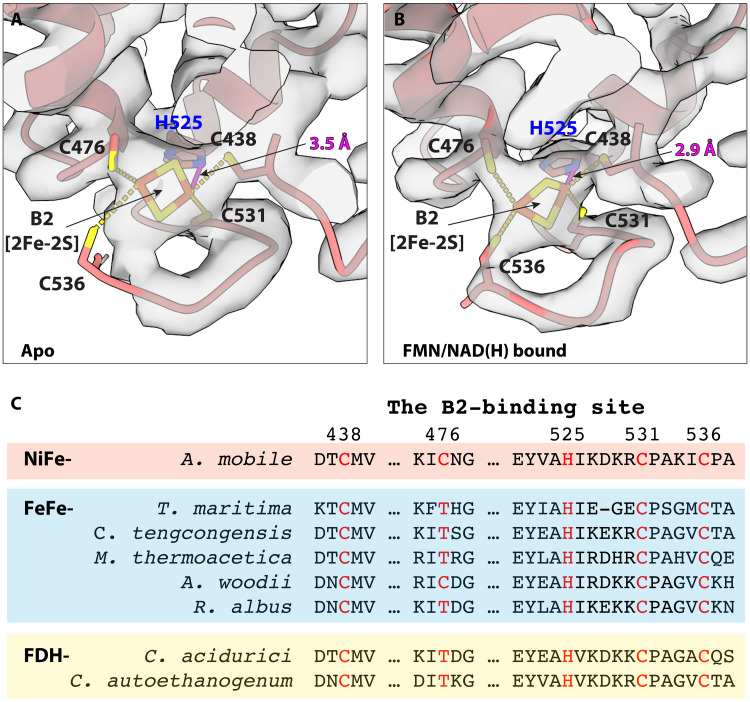
Comparison of the NiFe-HydB B2 [2Fe-2S] cluster coordination in the apo and NADH/FMN-bound state. (**A**) The five residues (4× Cys and 1× His) coordinating the B2 [2Fe-2S2] cluster are shown in sticks. (**B**) The B2 [2Fe-2S] moves toward His-525 in the NADH/FMN-bound state, likely due to a change in the ionization state of the histidine side chain. (**C**) Sequence alignment of several electron-bifurcating HydABC enzymes reveals the conservation of the B2-coordinating residues except for Cys^476^. NiFe-, FeFe-, and FDH- indicate that the enzymes are nickel-iron hydrogenase, iron-iron hydrogenases, and formate dehydrogenases, respectively.

In the FMN-free apo state structure of NiFe-HydABCSL, the distances between the C1 and B2 [2Fe-2S] clusters (19 Å) and between B2 and the B3 [4Fe-4S] clusters (18 Å) are too far to transfer electrons (Fig. 1E). Substantial conformational changes must therefore occur when FMN and/or NAD bind to enable electron transfer from FMN to B3, and this is discussed below. Notably, the B2 [2Fe-2S] cluster has an unusual environment as it is coordinated not only by four cysteines (Cys^438^, Cys^476^, Cys^531^, and Cys^536^) but also by His^525^ (Fig. 3, A and B). Three cysteines (except Cys^476^) and the His residues are conserved in the HydB subunit of the bifurcating FeFe-hydrogenases, while Cys^476^ is semi-conserved (either Cys or Thr) (Fig. 3C). Therefore, this unique coordination likely functions to modulate the redox potential for either storing or transferring low-potential high-energy electrons (see below). A recent preprint assigned a zinc ion in inactive *T. maritima* FeFe-HydABC expressed in *Escherichia coli* where we assigned the B2 [2Fe-2S] cluster (*23*). However, a Zn ion was not found in the functional FeFe-HydABC we previously purified from the native organism (*32*). Further investigations are needed to understand the cofactor content of the *T. maritima* enzyme.

### FMN coordination and NAD binding to HydB in NiFe-HydABCSL

**Fig. 4.**
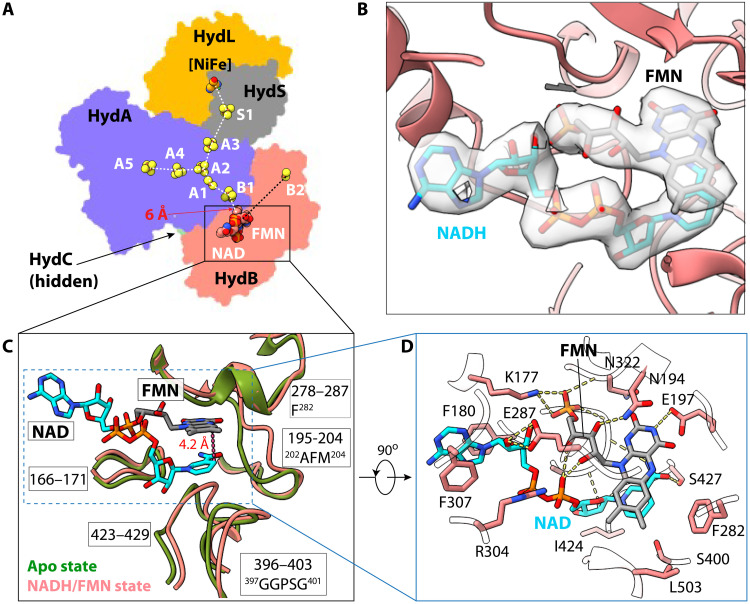
Structure of the NADH- and FMN-bound NiFe-HydABCSL. (**A**) Location of cofactors including FMN and NAD(H) in the overall HydABCSL architecture. The white dashed lines connect cofactors with distances <14 Å, and the black line connects cofactors >14 Å apart. (**B**) EM densities of NAD and FMN superimposed on the atomic model. (**C**) Key HydB loops coordinating FMN and NADH. These loops in the apo state (dark green) are superimposed with those in the FMN/NAD-bound state (salmon) to show conformational changes. The starting and end residue numbers of the five coordinating loops are shown. The amino acid sequence of the three motifs that distinguish the bifurcating hydrogenase from nonbifurcating versions is labeled. The 4.2-Å distance between the NAD nicotinamide C4 atom and the ionizable N5 atom in the FMN isoalloxazine ring is labeled. (**D**) Key HydB residues within 4 Å of FMN and NAD are shown in sticks and labeled. Hydrogen bonds between enzyme and cofactors are shown as dashed yellow lines.

To understand the electron transfer pathways in the NiFe-enzyme, we prepared cryo-EM grids in the presence of purified *A. mobile* Fd, FMN, and NADH. We obtained a cryo-EM 3D map at 3.0-Å average resolution with FMN and NADH bound (Fig. 4A and figs. S12 and S13). Fd was not resolved in the 3D map, perhaps because Fd binds only transiently. The structure is similar to that of the apo state, but the HydC Fd-like CTD and the HydB NTD, which were well resolved in the flavin-free apo state, became invisible in the high-resolution EM map. It is likely that the FMN and NADH binding caused a series of structural changes leading to the release of HydB NTD and HydC CTD from the main body of the structure and becoming flexible. Domain movement in response to cofactor binding is also observed in the evolutionarily unrelated bifurcating ETF family (*3*, *6*, *7*) and suggests that in the NiFe-enzyme, the flexibility of the HydC CTD might control electron transfer along the endergonic pathway to Fd.

FMN and NADH had well-defined densities in HydB (Fig. 4B) and are stacked in parallel to each other in a configuration that resembles those in Nqo1 of complex I (Fig. 2C) (*31*). Five signature motifs surround the FMN and NAD binding pocket: loops 166–171, 195–204, 278–287, 396–403, and 423–429 (Fig. 4, C and D). Three of these loops contain motifs that are distinct in bifurcating NiFe and FeFe-hydrogenases [the alanine, phenylalanine, methionine (AFM) motif in loop 195–204, F^282^ in loop 278–287, and the GGPSG motif in loop 396–403 in the NiFe-enzyme] as compared to nonbifurcating homologous complexes (*38*), while the other two loops are conserved across both BF and non-BF complexes (loops 166–171 and 423–429 in the NiFe-enzyme) (fig. S14). Compared to the apo state, all the FMN/NAD-coordinating loops underwent substantial conformational changes to accommodate the two cofactors. The distance between the NAD nicotinamide C4 atom and the ionizable N5 atom in the FMN isoalloxazine ring is only 4.2 Å, well within the hydride transfer range. Specifically, Lys^177^, Asn^194^, and Glu^197^ bind FMN via electrostatic interactions, and Phe^180^ and Phe^307^ sandwich the adenine ring, while Glu^287^ forms a hydrogen-bond with the ribose moiety (Fig. 4D). The FMN isoalloxazine ring is 6.0 Å from the B1 [4Fe-4S] cluster, providing an electron transfer path from FMN and the NiFe site via the chain of five clusters B1-A1-A2-A3-S1 (Fig. 4A).

Except for the three distinct motifs mentioned above, other residues in the FMN/NADH binding site are largely conserved (fig. S15). Because it sits at the meeting point of the NiFe-S1-A3-A2-A1-B1 and B5-B4-B3-B2-C1 pathways (Figs. 1E and 4A), the single-flavin FMN must play, at least in part, the role of electron bifurcation.

### Conformational changes are key to the bifurcation mechanism of NiFe-HydABCSL

**Fig. 5.**
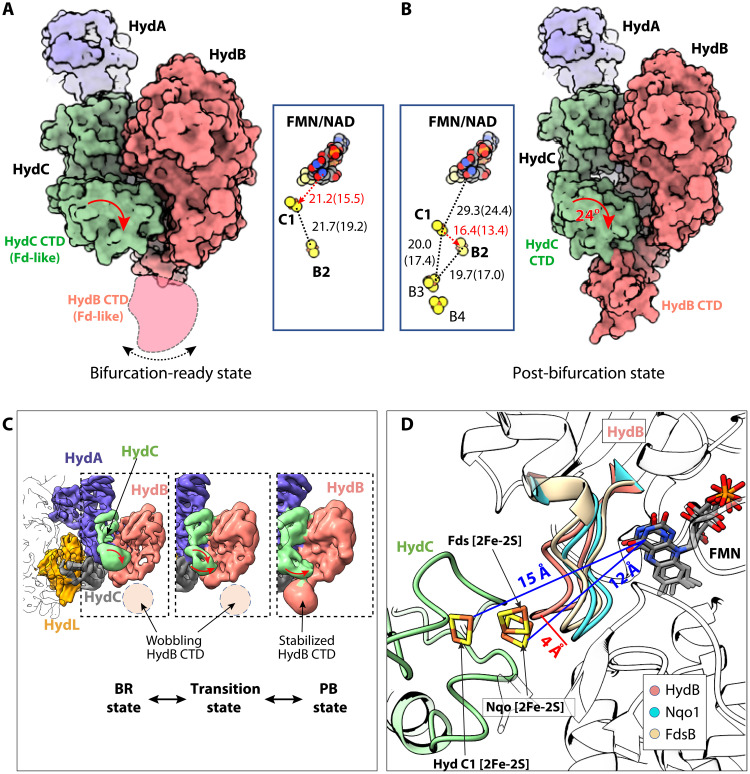
Two conformations of the NAD/FMN-bound NiFe-HydABCSL. (**A**) The first conformation is in a bifurcation-ready BR state, because the distance between FMN and the C1 cluster is nearly within the electron transfer range (red dashed line). The HydB Fd-like CTD is flexible in this conformation, as indicated by the double-headed black curve. (**B**) The second conformation is in a post-bifurcation PB state, because the distance between C1 and B2 clusters is nearly within the electron transfer range (red dashed line). Insets in both panels show the arrangement and distances between the resolved cofactors. The curved red arrows indicate a 24° rotation of the HydC Fd-like CTD between these two states. (**C**) The 1st (BR state), 10th (transition state), and 20th (PB state) EM density maps extracted from the 20-frame video derived from variability analysis. (**D**) The true B-state in which C1 [2Fe-2S] is 12 Å from FMN can be reached by a 4-Å movement of the FMN-binding HydB loop (amino acids 195 to 204, salmon), based on alignment with the Nqo (cyan, PDB ID 3IAM) and Fds structures (wheat, PDB ID 6TGA).

To gain insight into the conformational dynamics, we applied 3D variability analysis (*39*) and found that main dynamics is a correlated motion between the two Fd-like CTDs, one in HydC containing the C1 [2Fe-2S] cluster and one in HydB containing the B3 and B4 [4Fe-4S] clusters (Fig. 5 and movie S1). In the NAD/FMN-bound state, a fraction of the enzyme can be observed with a flexible HydB CTD, as seen in the averaged apo structure described above. The distance between the C1 [2Fe-2S] and the FMN isoalloxazine ring is 15.5 Å, approaching the 14-Å electron transfer distance (Fig. 5A). We have referred to this conformation as the “bifurcation-ready” or BR state, because the HydC CTD is poised to donate or accept a high-energy electron to or from FMN. We aligned NiFe-HydB, complex I Nqo1, and FDH FdsB structures around their shared FMN molecules and found that the 15.5-Å distance between FMN and B1 can be readily shortened to 12 Å for electron transfer if the second FMN-binding loop (loop 195–204) moves 4 Å closer to FMN as seen in the homologous structures (Fig. 5D). Therefore, we probably captured a stable intermediate stage of the NiFe-enzyme, and only a minimal conformational change would be needed to allow electron transfer between the C1 [2Fe-2S] cluster and FMN. The coordinated movement of HydC might act as a gate for low-potential electrons to prevent “short circuiting.” The BR state is likely coordinated with electron transfer from NADH in the forward reaction (production of H_2_). In the other end of the conformational movement of the 3D variability, the HydC CTD rotates by 24° and tilts down toward the B3 and B4 clusters such that the latter become stabilized (Fig. 5, B and C). The HydC CTD and HydB CTD are close enough that the distance between the C1 [2Fe-2S] and B2 [2Fe-2S] clusters is only 13.4 Å, well within the electron transfer range (Fig. 5B). Therefore, we have referred to this conformation as the “post-bifurcation” state (PB), because the downstream electron transfer distance between B2 and B3 (17.0 Å) or between C1 and B3 (17.4 Å) is slightly outside the range of electron transfer. It is likely that upon interaction with Fd, the HydB CTD will move to shorten these distances. The HydB NTD that contains B5 was not resolved in any FMN/NADH-bound state. The HydC CTD position is stabilized by the HydB NTD in the FMN-free apo state, and the movement of the HydC CTD during the bifurcation process needs the HydB NTD to be released. It is likely that the HydB NTD remains flexible during electron bifurcation.

### An electron transfer model of NiFe-ABCSL

**Fig. 6.**
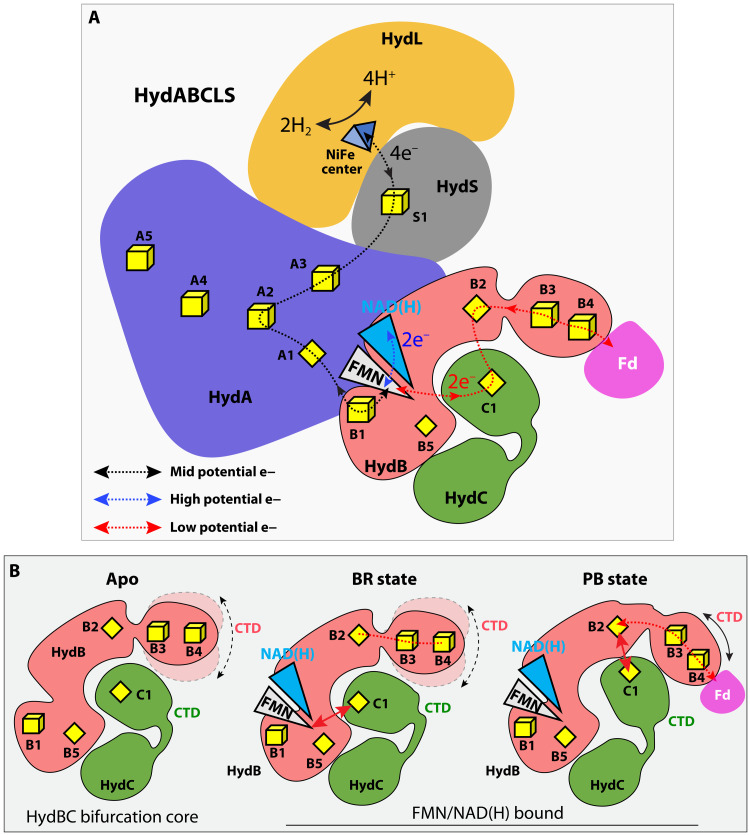
Proposed mechanism of electron bifurcation/confurcation in *A. mobile* NiFe-HydABCSL. (**A**) Overall electron transfer pathway, highlighting the three branches of the electron transfer path. The mid-potential path is a black dashed line, the exergonic path is a blue dashed line, and the endergonic path is a red dashed line. (**B**) Conformational changes in the HydBC bifurcation core from the electron bifurcation state (BR state) to the electron transduction state (PB state). See Results for details.

Our structural analyses suggest the following electron transfer pathways involving a minimum set of four cofactors—FMN, B1, B2, and C1 (Fig. 6, A and B). The enzyme is capable of bifurcation (H_2_ oxidation coupled to reduction of NAD and Fd) or the reverse reaction of confurcation. In the case of confurcation (Eq. 1), the enzyme evolves two molecules of H_2_ using higher potential electrons from NADH and lower potential electrons from reduced Fd. What is clear from the structure is that the A2-A4-A5 branch in HydA is a “nonfunctional” evolutionary remnant in terms of electron transfer, and the same appears to be true of the B5 cluster, although it is interesting that each is part of a Y arrangement of FeS clusters that might still have some as yet unknown function [see (*25*)]. The extended chain of FMN-B1-A1-A2-A3-S1-[NiFe] must transfer a total of four electrons from FMN to the [NiFe] site, two electrons originating from NADH and two electrons originating from Fd_red_. Hence, the electron path Fd-(B4-B3)-B2-C1-FMN is the endergonic branch. The donor Fd_red_ is expected to bind to the HydB Fd-like CTD, but this is not resolved in our EM structure, and we do not know whether the low-potential electrons are directly transferred from the B2 [2Fe-2S] cluster to Fd or if they are relayed via the B3 or B3 and B4 [4Fe-4S] clusters. We therefore find that conformational dynamics plays a crucial role in electron bifurcation in the NiFe-HydABCSL hydrogenase. The dynamics are largely confined to the Fd-like CTDs of HydB and HydC. In the absence of NAD and FMN, the HydB CTD is disordered, while the HydC CTD is stable (Fig. 6B). In the active state when both NAD and FMN are bound, we found that HydC CTD becomes mobile and interconverts between a bifurcation-ready BR state where electron transfer from FMN to the C1 [2Fe-2S] is permitted and a post-bifurcation PB state where electron transfer from C1 to B2 [2Fe-2S] is permitted.

The FMN of NiFe-HydABCSL is unique among the bifurcating flavins found in the other three families of bifurcating enzymes, all of which use FAD (*40*), although the significance of FMN versus FAD is not known. The FMN in the NiFe-enzyme accepts two electrons in a hydride transfer from NADH, but from the structure, the flavin is also in the pathway of electron transfer from reduced Fd_red_ to the NiFe site (Fig. 6A). Hence, this flavin must serve both a “bifurcation” role, where it is at the meeting point of three electron transfer pathways (from or to NADH, Fd_red_, and H_2_), and an electron transfer role, as all four electrons that reduce four protons to two H_2_ molecules must pass through FMN. In all other bifurcating enzymes, the BF-FAD accepts electrons from a mid-potential donor and is at the center of diverging high- and low-potential pathways (*1*), but that is not the case with the FMN in NiFe-HydABCSL. Moreover, it is also not clear how the FMN activates both electrons from NADH to more negative potentials so that they can reduce protons. We propose that it is a combination of FMN and the unique arrangement of clusters, B1, B2, and C1, that achieve bifurcation and the transfer of four electrons from two donors (NADH and Fd_red_). This is facilitated by conformational changes of HydC, and the His-coordinated B2 2Fe-cluster likely plays a key role. Detailed spectroscopic analyses will be required to elucidate the role of this unique FMN in the proposed pathways of electron transfer that are evident from the cryo-EM structure, and these are underway (Fig. 6A). However, the marked conformational changes observed in the HydBC bifurcation core must have enabled the FMN to serve two roles.

In summary, we have described the cryo-EM structure of NiFe-HydABCSL, an electron-bifurcating hydrogenase consisting of a conventional NiFe-hydrogenase module (SL subunits) and of a bifurcating module (ABC subunits) that is highly similar to a previously characterized bifurcating heterotrimeric FeFe-hydrogenase (*12*). Two other types of bifurcating complexes that contain the ABC module have been characterized, FDH and NADH-dependent NADPH (reduced form of nicotinamide adenine dinucleotide phosphate) Fd oxidoreductase (*19*, *41*), which use formate and NADPH, respectively, rather than H_2_ as electron donors for NAD and Fd reduction. Our work explains how different functional units, such as FDH and NiFe- and FeFe-based hydrogenases, can be coupled to the NAD- and Fd-dependent bifurcation reaction by connecting these units to the conserved ABC bifurcation core network containing Y-shaped FeS clusters in HydA that feed electrons to the bifurcating site, FMN. The NiFe-HydABCSL structures have revealed the importance of two [2Fe-2S] clusters in the bifurcating reaction, C1 in HydC and the uniquely coordinated B2 in HydB. This “4Cys + His” coordination might enable tuning of the redox potential of the B2 cluster. We suggest that the pathways of electron transfer proposed here are applicable to all members of bifurcating complexes that contain homologous HydABC domains, although the exact role of the enigmatic FMN in these enzymes remains unsolved.

## METHODS

### Microorganism

*A. mobile* ATCC BAA-54 (DSM 13181) was obtained from the German Collection of Microorganisms and Cell Cultures (DSMZ; Braunschweig, Germany).

### Growth of microorganisms

*A. mobile* was grown in a medium containing fructose (5 g/liter), yeast extract (0.5 g/liter), cysteine (1 g/liter), sodium bicarbonate (1 g/liter), 10 mM Mops, 1 mM KH_2_PO_4_, 9.3 mM NH_4_Cl, 4.4 mM KCl, 1.6 mM MgCl_2_.6H_2_O, 1.0 mM CaCl_2_.2H_2_O, 1× vitamin mix, and 1× trace elements. The 1000× vitamin mix contains, per liter, 4.0 mg of biotin, 4.0 mg of folic acid, 20 mg of pyridoxin-HCl, 10 mg of thiamin-HCl, 10 mg of riboflavin, 10 mg of nicotinic acid, 10 mg of d-Ca-pantothenate, 0.2 mg of vitamin B12, and 10 mg of *p*-aminobenzoic acid. The 1000× trace elements mix contains, per liter, 10 ml of 25% HCl solution, 1.50 g of FeCl_2_·4H_2_O, 70 mg of ZnCl_2_, 100 mg of MnCl_2_·4H_2_O, 6.0 mg of H_3_BO_3_, 190 mg of CoCl_2_·6H_2_O, 2.0 mg of CuCl_2_·2H_2_O, and 24 mg of NiCl_2_·6H_2_O. The pH of the medium was adjusted to pH 7.0 and filter-sterilized using a 0.2-μm filter. *A. mobile* was routinely grown anaerobically in 100-ml serum bottles with 50 ml of medium and a headspace of 20% CO_2_ and 80% N_2_ at 55°C with shaking (120 rpm). When grown in the 20-liter fermenter, an additional peptone (10 g/liter) from meat extract and V8 juice (2 ml/liter; Campbell Soup Co., Chicago, IL) was added to the medium. The fermenter was stirred at 150 rpm and was sparged with 20% CO_2_ and 80% N_2_ at a rate of 1.5 liters/min. *A. mobile* cells were harvested by centrifugation (10,000*g* in a continuous-flow Sharples centrifuge) and were subsequently frozen in liquid N_2_ and stored at −80°C.

### Purification of *A. mobile* NiFe-hydrogenase and Fd

All the following protein purification steps were performed anaerobically either in a Coy anaerobic chamber (95% Ar, 5% H_2_) or using sealed serum bottles with an Ar headspace using buffers degassed with Ar under positive pressure with a constant flow of Ar. Cells (20 g wet weight) were resuspended in 100 ml of buffer [50 mM Hepes (pH 7.5), 5% glycerol, 5% trehalose, 1 mM cysteine, 0.1 mM phenylmethylsulfonyl fluoride, and deoxyribonuclease (50 mg/liter)]. Cells were broken on ice by sonication (30-s intervals, amplitude 60; Qsonica, model Q55). An extract of soluble proteins was prepared by ultracentrifugation at 100,000*g* for 1 hour. Anion exchange chromatography was carried out using a 50-ml QHP custom column (XK 26/20 Cytiva, Marlborough, MA) equilibrated with 50 mM tris (pH 8.0), containing 5% glycerol, 2.5% trehalose, and 1 mM cysteine. Bound proteins were eluted with a linear gradient for 0 to 500 mM NaCl in the same buffer; the hydrogenase activity was eluted as a single peak when ~300 mM NaCl was applied. Size exclusion chromatography was carried out using a HiLoad Superdex 200 prepgrade XK 16/60 column (Cytiva, Marlborough, MA) with a running buffer of 25 mM tris (pH 8.0), with 300 mM NaCl, 5% glycerol, and 2.5% trehalose at a flow rate of 1.25 ml/min. All fractions were collected in Ar-flushed serum vials sealed with butyl rubber stoppers and were stored at 4°C. *A. mobile* Fd was purified from the same batch of cells; it eluted from the anion exchange column when 250 mM NaCl was applied. In the size exclusion step, the Fd eluted around 10 kDa from the HiLoad Superdex 200 XK 16/60 column (fig. S3A) based on the column ultraviolet-visible (UV-vis) monitoring and metal analysis.

### Hydrogenase analyses

H_2_ oxidation assays were performed in an Agilent UV-vis spectrometer by following the reduction of benzyl viologen at 600 nm (ε = 7400 cm^−1^ M^−1^) at 50°C. The reaction mixture (2.0 ml) contained 50 mM Hepes (pH 7.5), 1 mM benzyl viologen, ~4 μM sodium dithionite, and a 100% H_2_ headspace, and was started with enzyme (1 to 100 μg protein). Activity is reported in units where one unit catalyzes the reduction of 1 μmol of benzyl viologen per minute. Specific activity is calculated using protein estimations based on the Bradford method (*42*) using bovine serum albumin as the standard. For the bifurcating assay, the reduction of Fd was monitored at 425 nm (ε = 13 mM^−1^ cm^−1^). The reaction mixture (600 μl) contained a headspace of 100% H_2_, *A. mobile* Fd (40 μM), and 10 μM FMN. The reaction was initiated by the addition of 1 mM NAD. The units for the bifurcating hydrogenase reactions are defined as μmol H_2_ produced or oxidized per minute per milligram. The amount of FMN in the hydrogenase was determined by denaturing the protein sample (~1 mg/ml) by incubation with 1% SDS at 25°C for 10 min. The concentration of free FMN was calculated by the absorbance at 450 nm (ε = 12.2 mM^−1^ cm^−1^).

### Cryo-EM grid preparation and data collection

The purified NiFe-hydrogenase was aliquoted to prepare EM grids for single-particle cryo-EM analysis. The protein stock was stored in tightly stoppered vials to maintain the sample in an oxygen-free environment. The sample was withdrawn from the vial using 10-μl gastight syringe (model 1801, Hamilton) instead of micropipette to avoid the exposure to air as much as possible. Then, 4 μl of sample was applied to glow-discharged holey carbon grids (Quantifoil R2/1 Copper, 300 mesh) in the humidity chamber of FEI Vitrobot Mark IV. The EM grids were blotted for 3 s using a piece of filter paper and then plunged into liquid ethane cooled by liquid nitrogen. To prepare the NADH reduced state, the FMN solution (1 mM), NADH solution (1 mM), and Fd solution (500 μM) are quickly mixed with the protein solution with a final concentration of 100 μM FMN, 100 μM NADH, and 10 μM Fd. The sample was then applied to the grid in the same way. The pilot datasets of around 1000 micrographs were collected in a 200-kV FEI Arctica electron microscope equipped with a K2 summit camera (Gatan) for screening purposes. The 3D reconstruction and refinement led to preliminary 3D map around 5 Å to confirm the quality of the grids. The grids were then collected on a TFS Titan Krios electron microscope operated at 300 kV. The apo-state NiFe-HydABCSL was collected with a K2 summit camera (Gatan), and the NADH-reduced state was collected with K3 summit camera (Gatan) after an upgrade. The two datasets were acquired with the objective lens under-focus range of −1.0 to −2.0 μm at a nominal magnification of ×130,000 using SerialEM (*43*), with an effective calibrated image pixel size of 0.514 and 0.414 Å, respectively. All EM images were recorded in the super-resolution counting and movie mode with a dose rate of 1.9 (apo state) and 1.4 (FMN/NADH-bound state) electrons per Å^2^ per second per frame. A total of 40 frames were recorded in each movie micrograph.

### Image processing

The apo-state dataset and NADH-bound state dataset are processed in the same fashion. They contained 9858 and 8088 movie stacks, respectively. These movies were drift-corrected with electron-dose weighting and twofold binned using MotionCor2.1 (*44*). The full dataset was split into four subsets and imported to Relion-3 (*45*). For each subset, the particles were auto-picked and extracted with 4× binning. After 2D classification, the “good” 2D class averages with defined structural features were selected and imported into Cryosparc2 (*46*) for ab initio 3D model reconstruction. The particle images from the best 3D map with good structural details that were reconstructed from each of the four subsets were merged and converted to the RELION format using UCSF PyEM (https://github.com/asarnow/pyem). At this stage, the two datasets have 404,165 particles and 306,395 particles, respectively. Another round of 3D classification was run on the particles before C2-symmetry 3D refinement led to the 3.2-Å density map for the apo state and the 3.0-Å density map for the NADH-bound state, based on the 0.143 threshold of the gold standard Fourier shell correlation between the independently constructed 3D “half” maps, with each map using half of the dataset. The local resolution map was calculated using ResMap and displayed using UCSF Chimera.

### Model building and refinement

The maps were postprocessed with DeepEMhancer for better density map details (*47*). The initial atomic model for each chain in NiFe-HydABCSL was built with comparative modeling while fitting into the electron density map with the Rosetta suite (*48*). The templates are corresponding homologs in nonbifurcating hydrogenase Hox tetramer in *H. thermoluteolus* (PDB ID 5XF9) (*29*) except that the HydA subunit was built using the structure of NADH quinone oxidoreductase subunit 3 in *T. thermophilus* (PDB ID 3IAM) (*31*) as template. All the atomic models were then manually rebuilt or refined with the program COOT (*49*) followed by real-space refinement in the PHENIX program (*50*). Last, the atomic model was validated using MolProbity (*51*, *52*). The distance between different cofactors was calculated in PyMOL by the shortest atom distance (side-to-side) or the distance of geometric center (center-to-center).

The low-resolution regions of density map include the NTD and CTD in HydB and the Fd-like CTD of HydC in the FMN/NADH-bound state. For the NTD and CTD in HydB, the structure was modeled with online server Robetta (*34*) followed by a refinement in Phenix (*50*). The modeling of Fd-like CTD of HydC in different conformations can be found in the section of protein dynamics analysis. The figures were generated with PyMOL (The PyMOL Molecular Graphics System, version 1.8.x; Schrödinger) or UCSF ChimeraX (*53*).

### Protein dynamics analysis

Programs in cryoSPARC2 (version 3.1) (*46*) were used to analyze the conformational change observed in the dataset of FMN/NADH-bound NiFe-HydABCSL. Because the particles have C2 symmetry, the particle images were first expanded based on its symmetry to gain more information on single protomers. The 3D variability analysis (*39*) in cryoSPARC2 was then applied to only one protomer of the expanded particle images. Twenty frames (density maps) were generated, which demonstrated the movement of HydC CTD. The structural model of this domain from the flavin-free apo state was rigid-docked into each density map in each frame to generate the structural model for that frame. Then, the loop connecting NTD and CTD of HydC was rebuilt in COOT (*49*). Figures and movies were generated using UCSF ChimeraX (*53*).
