# Supplementary material for: Structure and electron transfer pathways of an electron-bifurcating NiFe-hydrogenase
Source: Sci Adv. 2022 Feb 25;8(8):eabm7546. doi: 10.1126/sciadv.abm7546 (PMC8880783; doi:10.1126/sciadv.abm7546)
Supplement: Supplementary file 1 — Table S1 Figs. S1 to S15 [file sciadv.abm7546_sm.pdf]

Science Advances
AAAS

Supplementary Materials for

Structure and electron transfer pathways of an electron-bifurcating NiFe-hydrogenase

Xiang Feng, Gerrit J. Schut, Dominik K. Haja, Michael W. W. Adams*, Huilin Li*

*Corresponding author. Email: adamsm@uga.edu (M.W.W.A.); huilin.li@vai.org (H.L.)

Published 25 February 2022, *Sci. Adv.* **8**, eabm7546 (2022)
DOI: 10.1126/sciadv.abm7546

The PDF file includes:

Table S1
Figs. S1 to S15
Legend for movie S1

Other Supplementary Material for this manuscript includes the following:

Movie S1

Supplementary Table 1. Cryo-EM data collection, refinement, and validation statistics for the *A. mobile* NiFe-HydABCSL

	NiFe-HydABCSL FMN free apo state (EMD-25633, PDB 7T2R)	NiFe-HydABCSL FMN/NAD(H) bound state (EMD-25647, PDB 7T30)
**Data collection and processing**		
Microscope	FEI Titan Krios	FEI Titan Krios
Voltage (kV)	300	300
Electron exposure (e^{-} Å^{-2})	76	56
Defocus range (μm)	-1.0 — -2.0	-1.0 — -2.0
Pixel size (Å)	1.029	0.828
Symmetry imposed	C2	C2
Initial particle images (no.)	404,165	306,395
Final particle images (no.)	269,151	217,361
Map resolution (Å)	3.2	3.0
FSC threshold	0.143	0.143
Map resolution range (Å)	3.2-6.0	3.0-6.0
**Refinement**		
Map sharpening B factor (Å)	-119.8	-103.1
Model composition		
Non-hydrogen atoms	29,216	28,126
Protein residues	3,736	3,578
R.m.s. deviations		
Bond lengths (Å)	0.010	0.006
Bond angles (°)	1.48	1.06
Validation		
MolProbity score	2.62	2.66
Clashscore	9.97	11.1
Poor rotamers (%)	5.23	6.1
Ramachandran plot		
Favored (%)	89.7	91.8
Allowed (%)	10.3	8.2
Outliers (%)	0.0	0.0

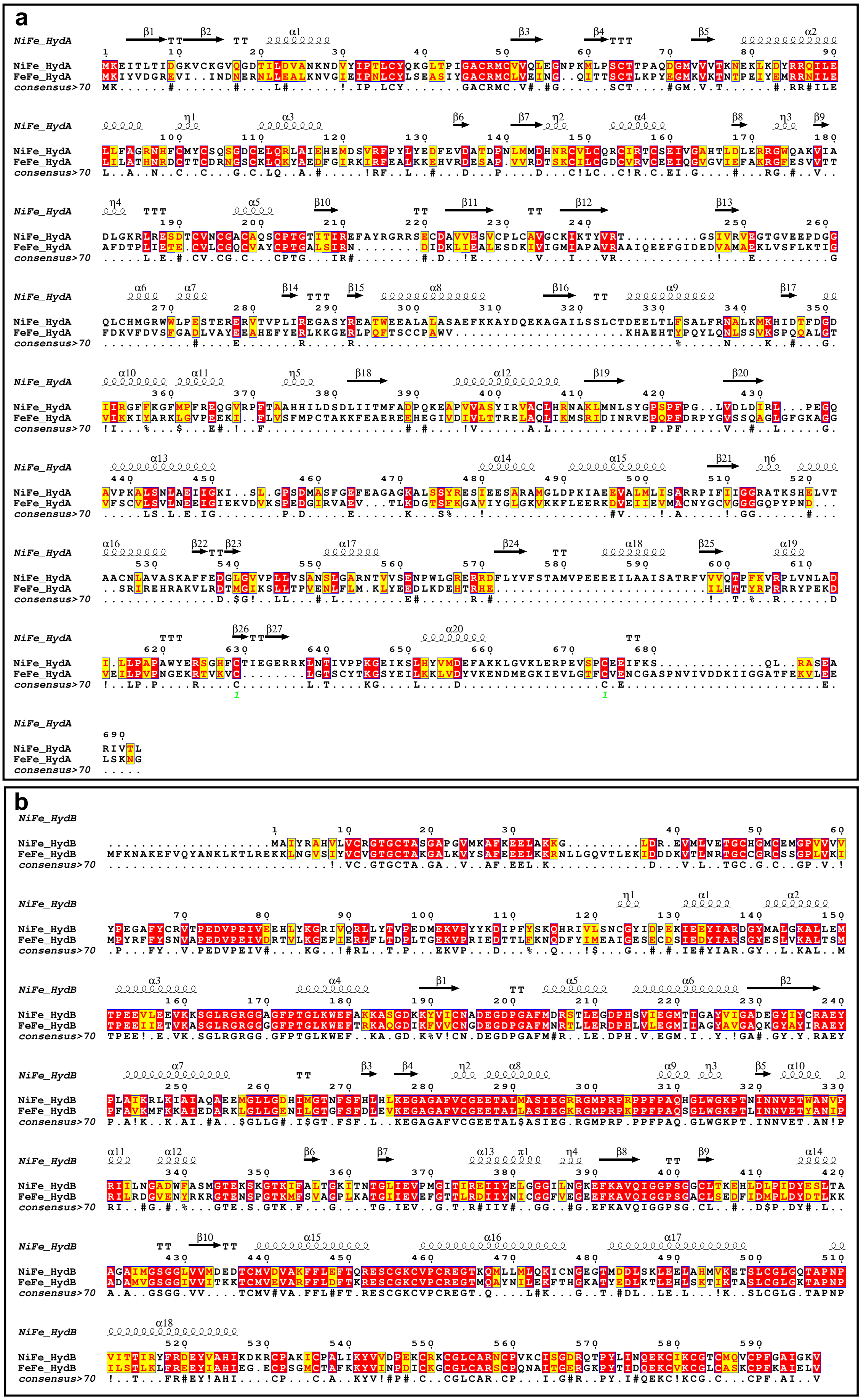
a
NiFe_HydA
FeFe_HydA
consensus>70
b
NiFe_HydB
FeFe_HydB
consensus>70

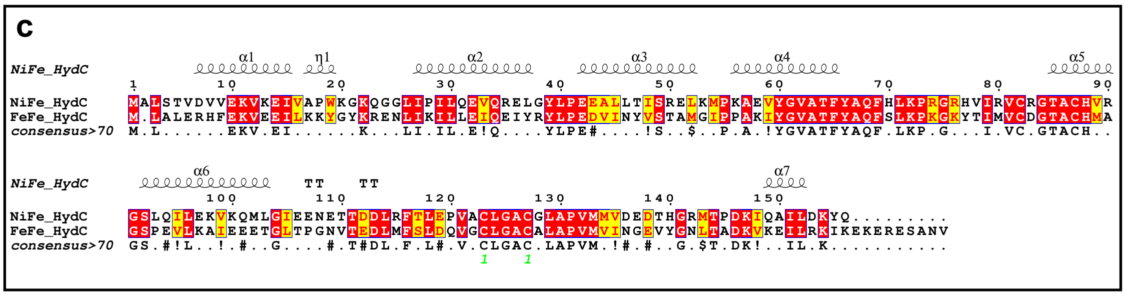

Supplementary Figure 1. Sequence alignment of HydA (a), HydB (b), and HydC (c) of *A. mobile* bifurcating NiFe-HydABCSL with the corresponding subunits of the *T. maritima* electron bifurcating FeFe-HydABC. The sequence identity is 21.4%, 54.3%, and 41.8%, respectively, for HydA, HydB, and HydC. The similarity between the two HydA proteins is low because the NiFe-HydA does not harbor the H-cluster in the CTD. However, the NTDs of the two HydAs are more similar with an identity of 32.9%.

a

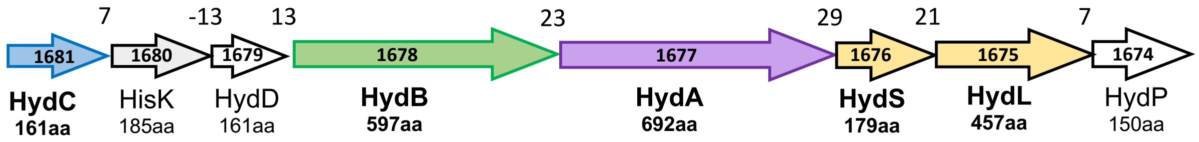

b

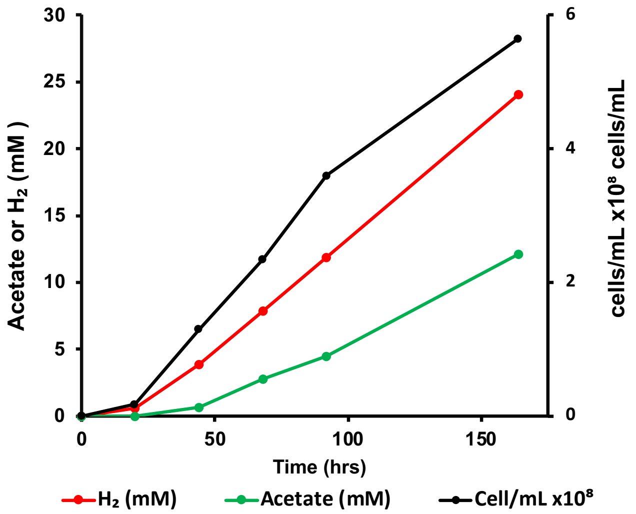

c

Step	Total Activity (U)	Yield (%)	Protein (mg)	Specific Activity (U/mg)
Cytoplasmic extract	9720	100	936	10.4
Q Sepharose	6455	66	121	25.2
Superdex S200	3132	32	4.1	261

d

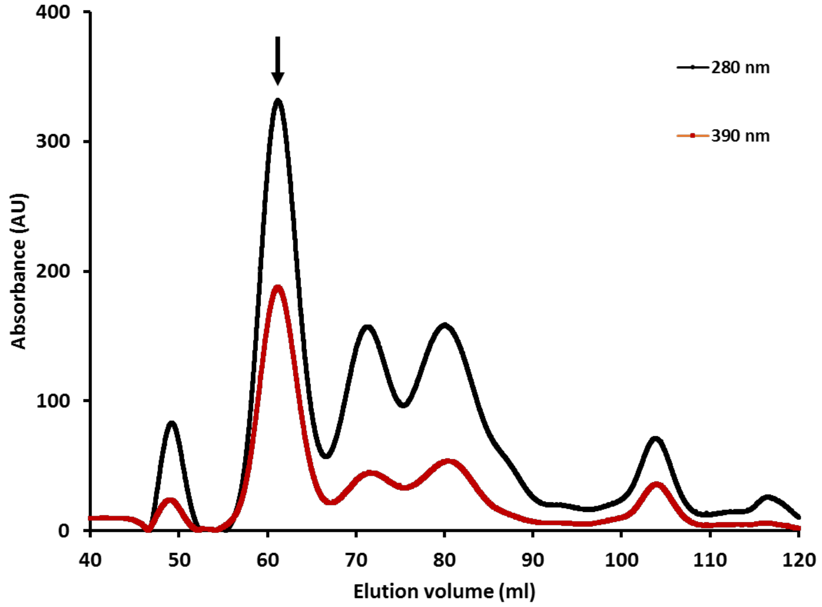

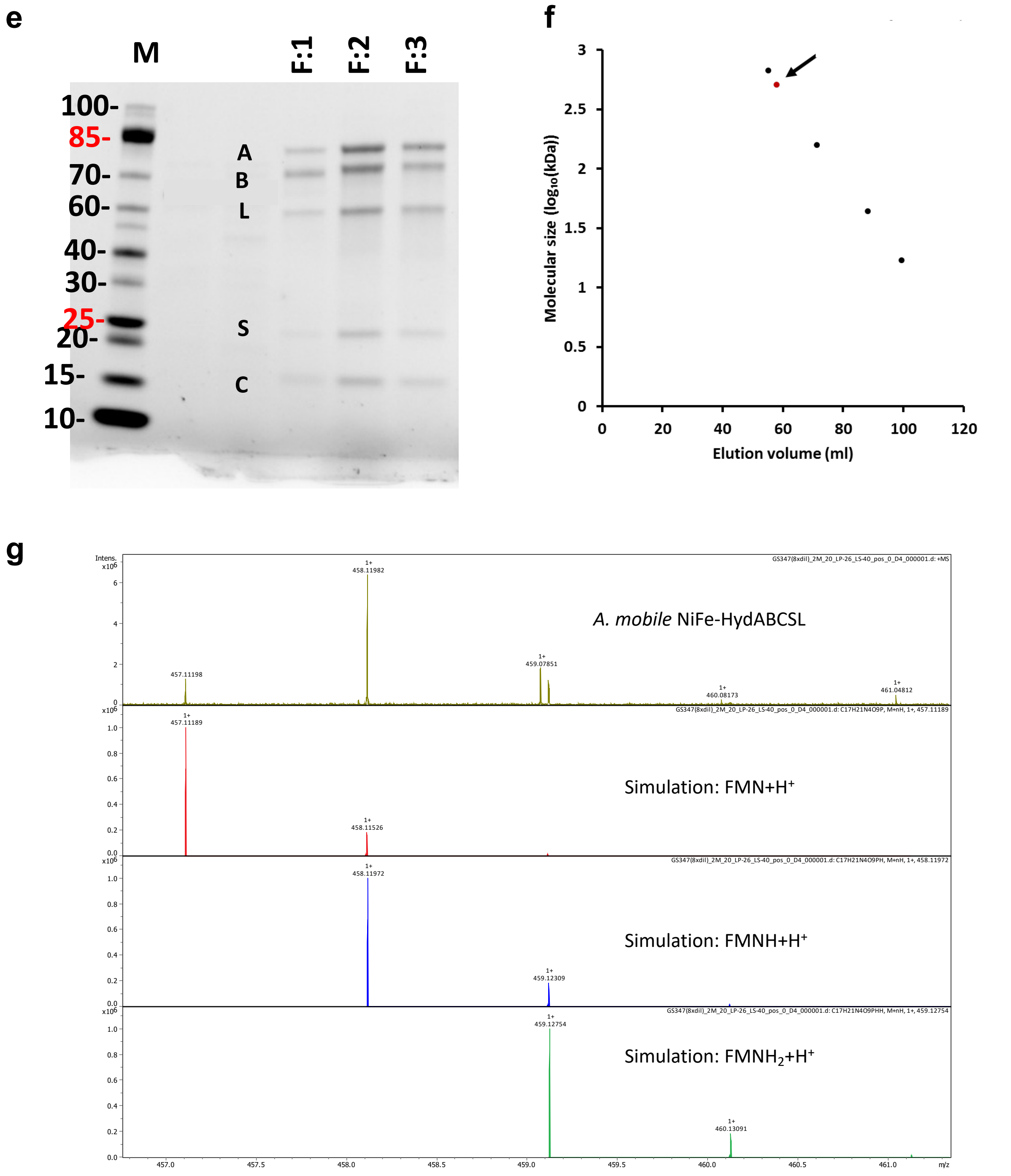

Supplementary Figure 2. Purification and characterization of *A. mobile* NiFe-HydABCLS. a) The operon containing the genes encoding the NiFe-HydABCSL hydrogenase. Gene numbers (Anamo_) and intergenic distances are indicated. **b**) Production of acetate and H_2 during growth of *A. mobile* on fructose. **c**) Purification of NiFe-HydABCLS where 1 U is 1 μmol of H_2 produced/min using reduced methyl viologen as the electron donor. **d**) Elution profile from the final Superdex S200 size exclusion

chromatography step in the purification of NiFe-HydABCSL. The vertical arrow points to the peak fractions used for SDS gel and structural analyses. Absorbance at 390 nm measures the iron sulfur cluster content present in the various protein peaks that are indicated by the absorbance at 280 nm. **e**) SDS-gel (Biorad, TGX 4-20% minigel) of purified hydrogenase fractions (F1-F3) from the final SEC step showing the five subunits (ABCSL). **f**) SEC elution profiles of NiFe-HydABCSL (red dot) and the molecular mass standards (black dots) bovine thyroglobulin (670 kDa), bovine γ-globulin (158 kDa), chicken ovalbumin (44 kDa) and horse myoglobin (17 kDa). **g)** Samples of purified NiFe-HydABSCL were spotted directly in a 2,5-dihydroxybenzoic acid matrix without digestion prior to analysis. The resulting spectrum is compared with the predicted spectra of the various ionization and redox states of FMN. The analysis was performed with a HR SolariX (Bruker) instrument using a MALDI source at the University of Georgia Proteomics and Mass Spectrometry Facility.

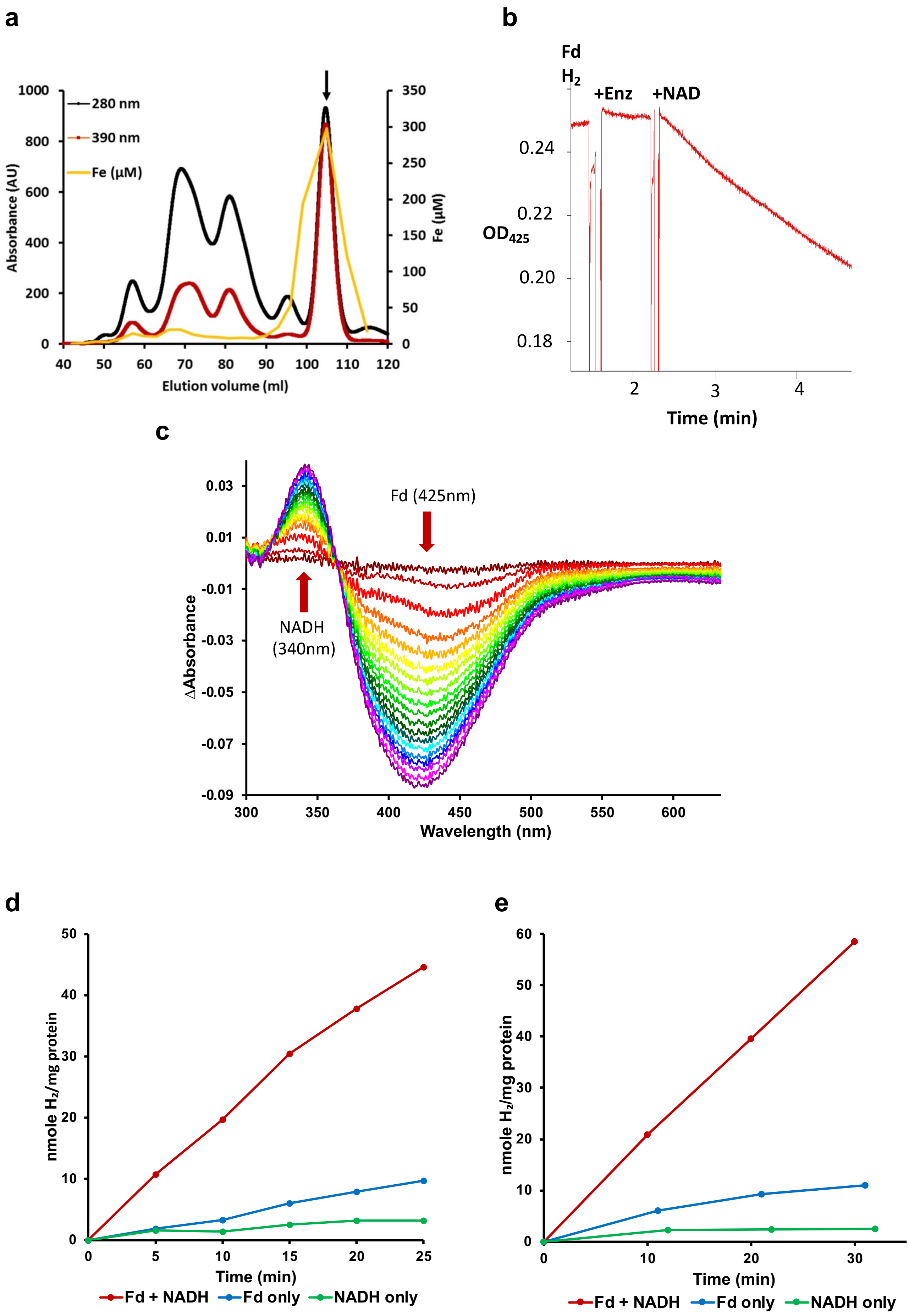

a
1000
800
600
400
200
0
Absorbance (AU)
280 nm
390 nm
Fe (μM)
350
300
250
200
150
100
50
0
Fe (μM)
40
50
60
70
80
90
100
110
120
Elution volume (ml)
b
Fd
H_2
+Enz
+NAD
0.24
0.22
OD_{425}
0.20
0.18
2
3
4
Time (min)
c
0.03
0.01
-0.01
-0.03
-0.05
-0.07
-0.09
ΔAbsorbance
Fd (425nm)
NADH
(340nm)
300
350
400
450
500
550
600
Wavelength (nm)
d
50
40
30
20
10
0
nmole H_2/mg protein
0
5
10
15
20
25
Time (min)
Fd + NADH
Fd only
NADH only
e
60
50
40
30
20
10
0
nmole H_2/mg protein
0
10
20
30
Time (min)
Fd + NADH
Fd only
NADH only

Supplementary Figure 3. Purification of *A. mobile* ferredoxin and the bifurcating activity of NiFe-HydABCSL. a) Purification of *A. mobile* ferredoxin using Superdex S200 size exclusion chromatography. The vertical arrow points to the peak ferredoxin-containing fraction used for enzyme assays and for the structural analysis. The absorbance at 390 nm indicates the presence of iron sulfur clusters. The Fe concentration was measured by ICPMS. **b)** Bifurcation activity of NiFe-HydABCSL as measured by Fd reduction (decrease in OD_{425}) was only observed when NAD (1 mM) was added to the reaction mixture (400 µl) of Fd (20 µM) under a H_2 atmosphere in the presence of the enzyme (Enz, 60 µg purified NiFe-HydABCSL). **c)** Simultaneous reduction of Fd (decrease in OD_{425}) and NAD (increase in OD_{340}) by the bifurcation activity of NiFe-HydABCSL. The UV-vis spectrum (300-700 nm upcycled Agilent HP 8453 operating under OlisWorks (Olis)) was recorded every 30 secs indicated by the rainbow scale lines. The reaction contained 25 µg NiFe-HydABCSL, 30 µM Fd, 1 mM NAD in 400 ul stoppered cuvettes under a H_2 atmosphere. The reduction of Fd and NAD are indicated by the red arrows at 425 and 340 nm, respectively. **d)** Bifurcation activity of the *A. mobile* cytoplasmic extract measured by H_2 production from NADH (green line), reduced Fd (blue line), or NADH and reduced Fd (red line). Assays were performed in 8 ml stoppered serum vials containing 50 mM HEPES pH 7.5 (1.0 ml), 500 µg cytosolic extract, 10 µM *A. mobile* Fd, 40 µg of pyruvate Fd oxidoreductase enriched fraction, 10 mM pyruvate, 0.5 mM TPP, 0.5 mM CoASH, 10 µM FMN and 1 mM NADH. Fd was kept reduced by added partially purified *A. mobile* pyruvate Fd oxidoreductase (POR, 40 µg of specific activity 12 µmole pyruvate oxidized/mg/min) and driven by the oxidation of pyruvate [12]. **e)** Bifurcation activity of purified NiFe-HydABCSL (70 µg) measured by H_2 production using the same reaction described above with the enzyme replacing the cytoplasmic extract

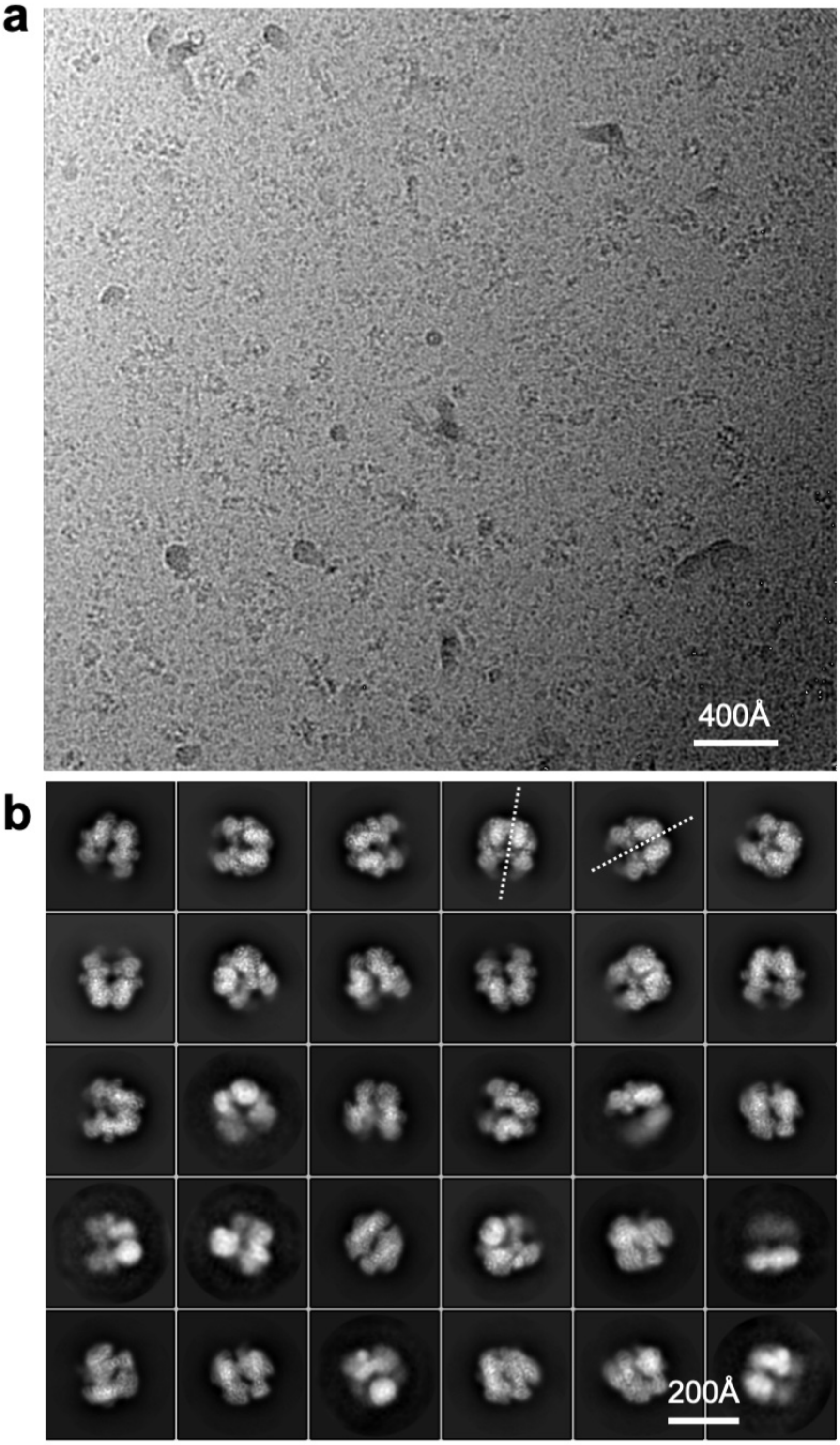

Supplementary Figure 4. Cryo-EM of the purified NiFe-HydABCSL. a) A typical raw micrograph after motion correction. **b**) 2D class averages. The dashed white lines indicate the presence of the mirror symmetry in some of the averages, indicative of the 2-fold symmetry of the complex.

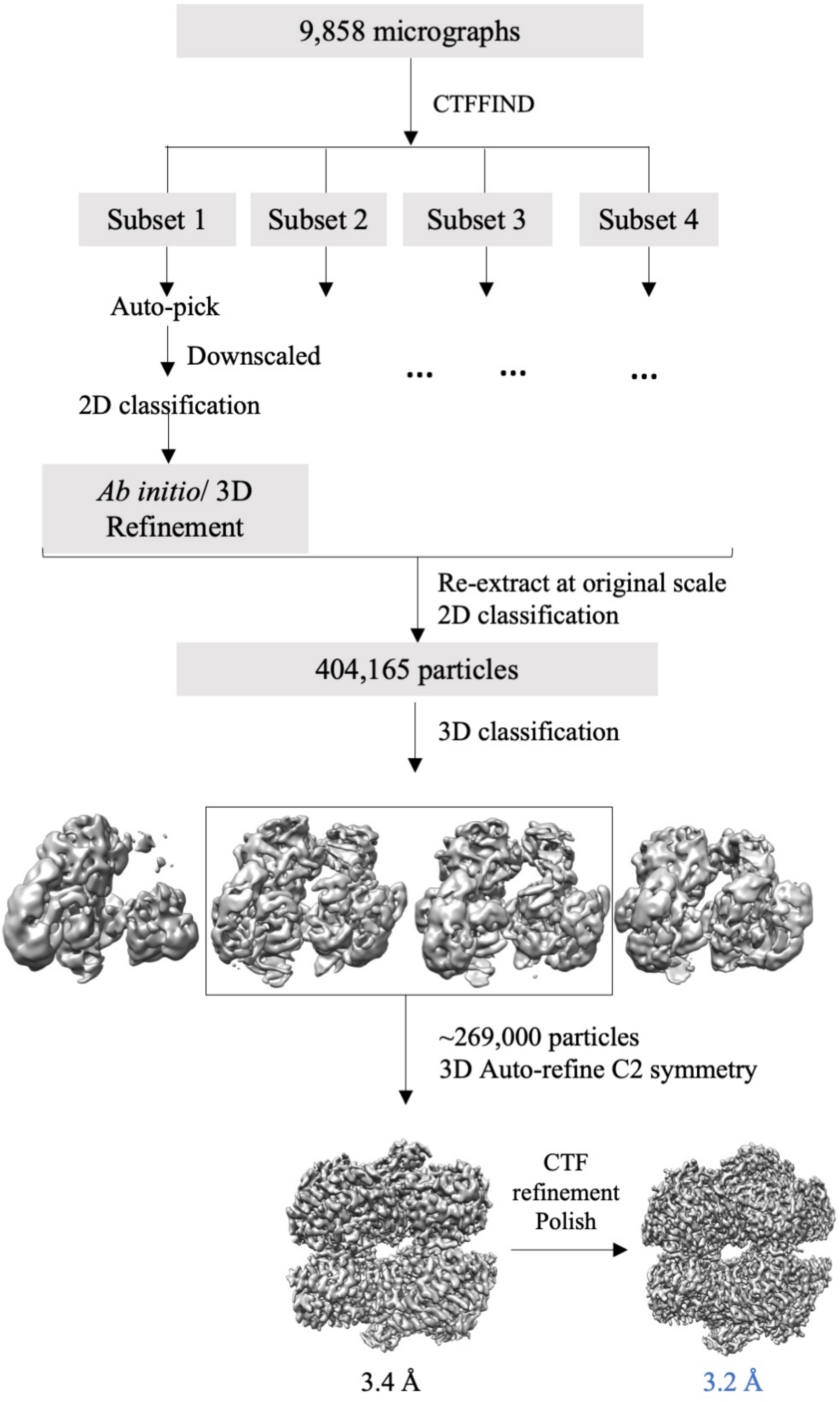

Supplementary Figure 5. Cryo-EM image processing and 3D reconstruction pipeline of the apo NiFe-HydABCSL. Note the 3D maps shown in the bottom row are in a different view than those 3D classification derived 3D maps.

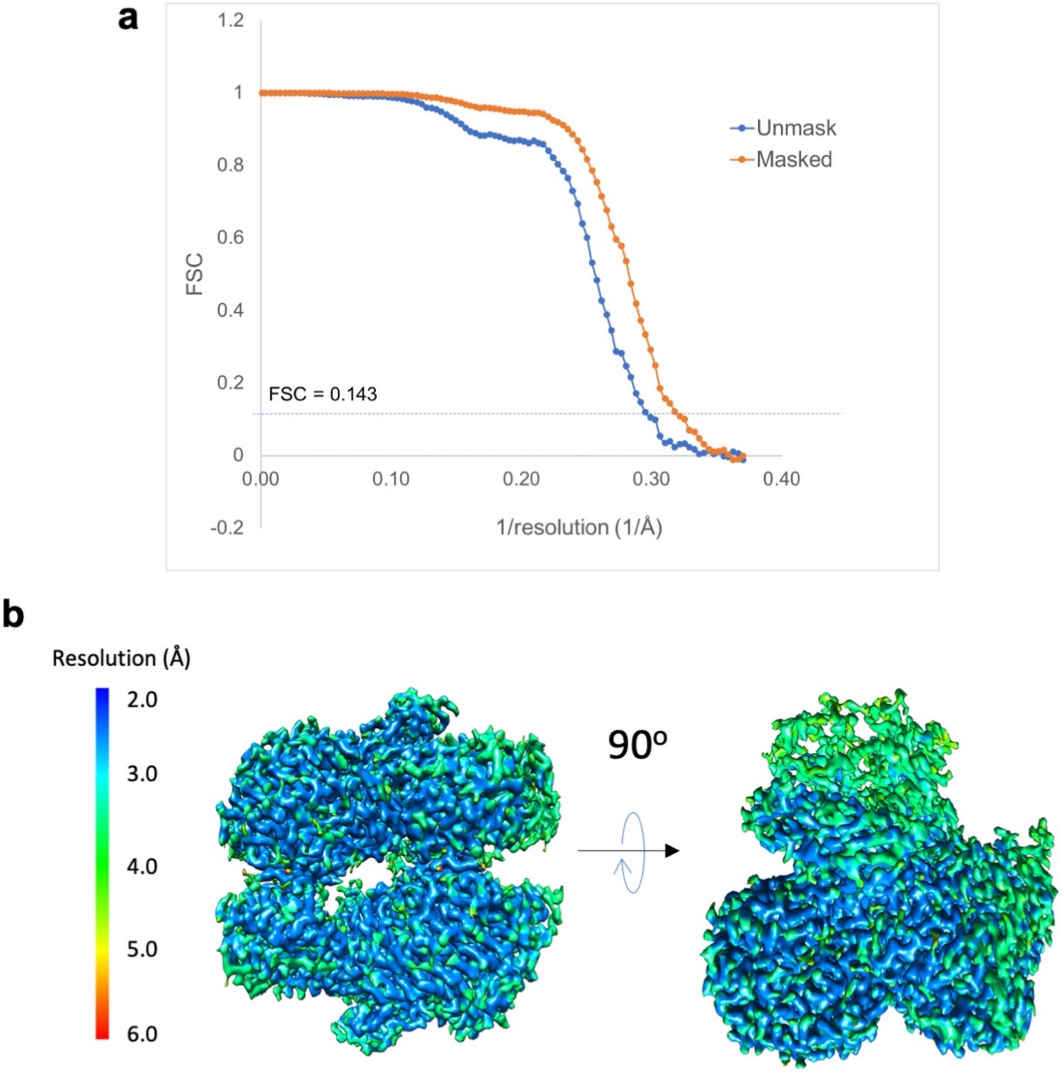

Supplementary Figure 6. Resolution estimation of the final 3D map. a) Fourier shell correlation curves of two half maps calculated with and without mask, respectively. The standard 01.43 correlation threshold was used. **b**) Color coded 3D local resolution map.

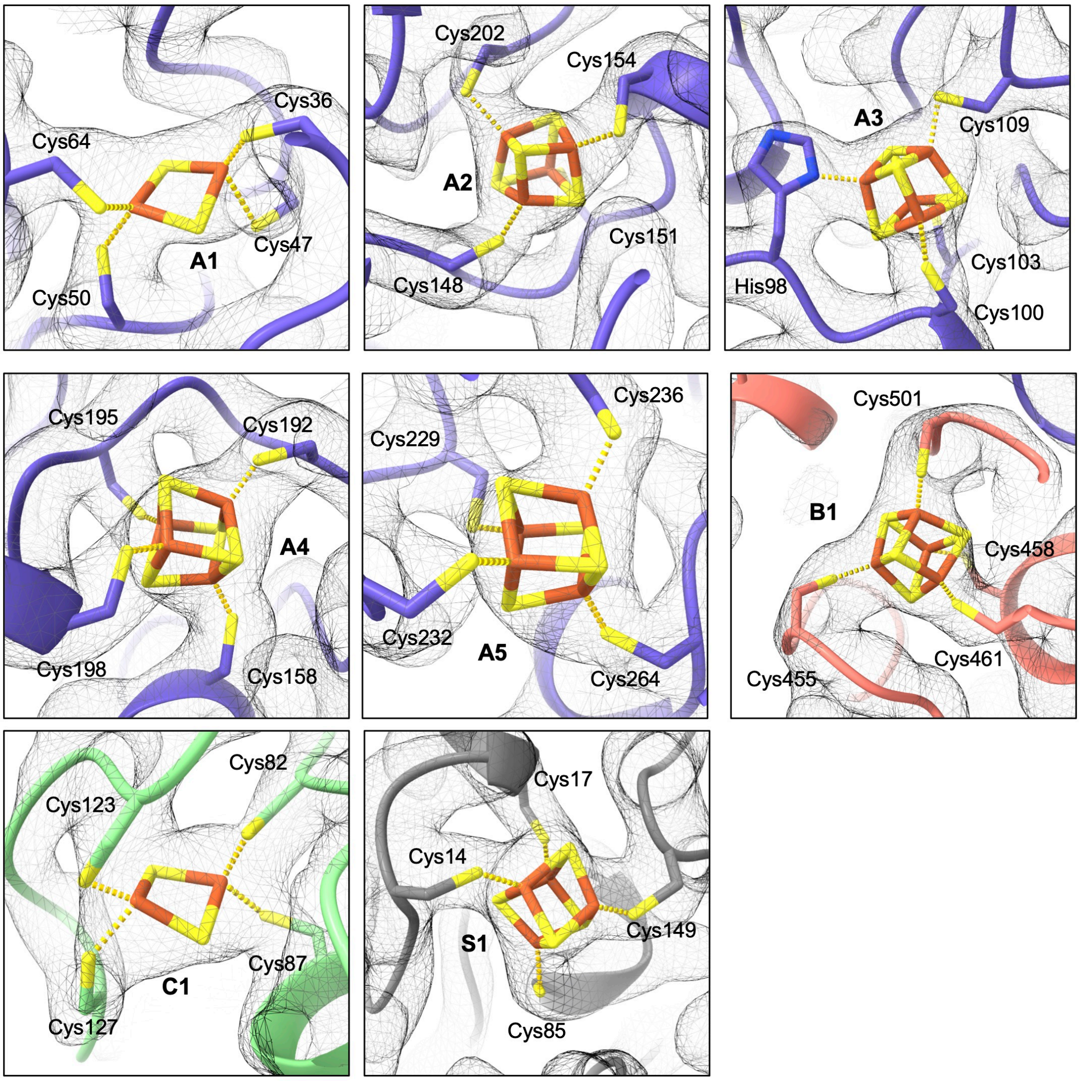

Supplementary Figure 7. EM densities of the Fe-S clusters surfaced rendered at a threshold of 4σ. The modeled clusters and their coordinating residues are shown in sticks. The EM densities of several co-factors are not shown here because they are presented elsewhere: FMN and NADH in Fig. 4, B2 in Fig. 3, B3, B4, and B5 in Supplementary Fig. 7.

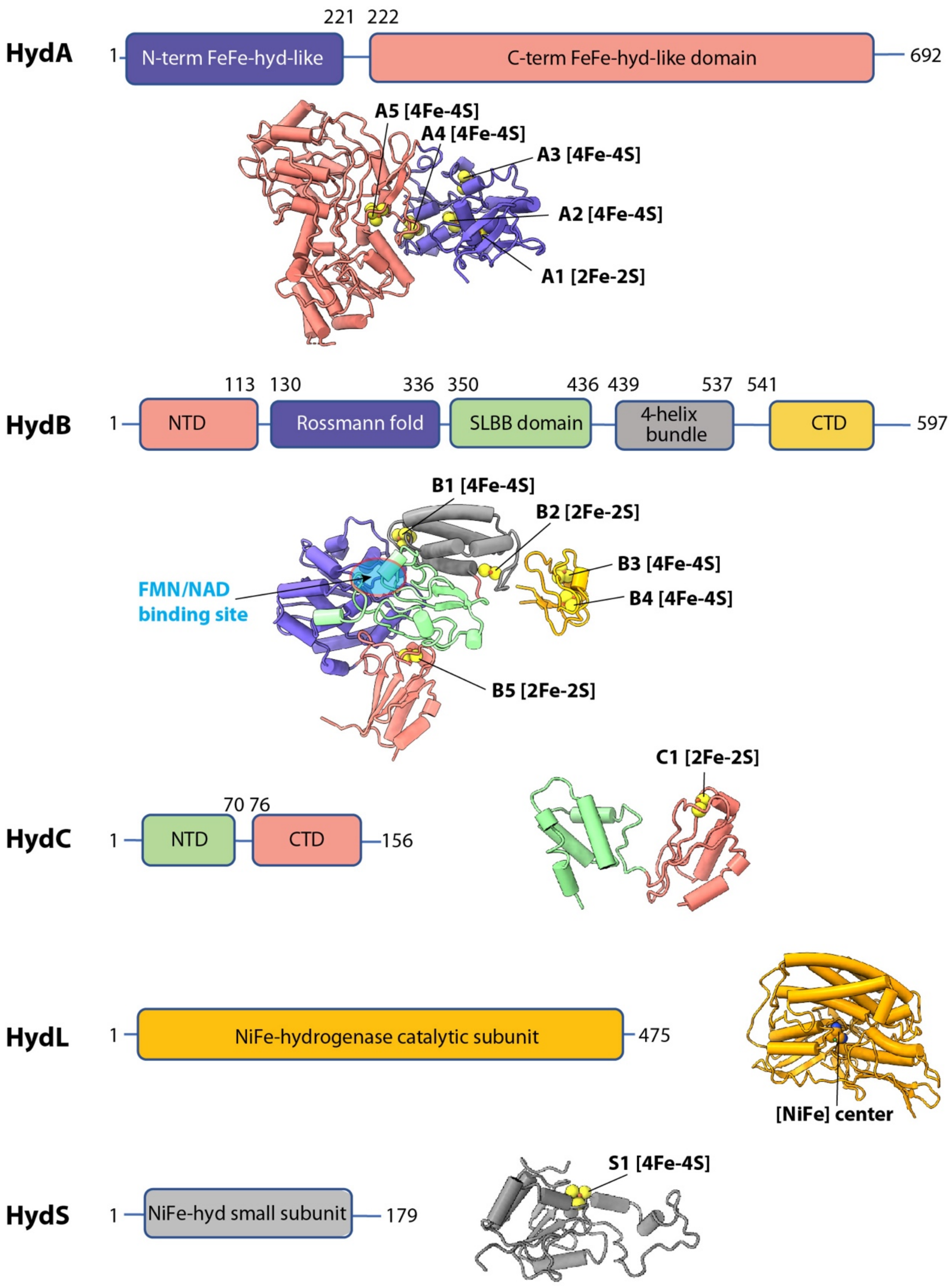

Supplementary Figure 8. Structures of HydABCLS subunits are shown separately in cartoon, with domains colored individually**.** The bound cofactors are shown in spheres and labeled.

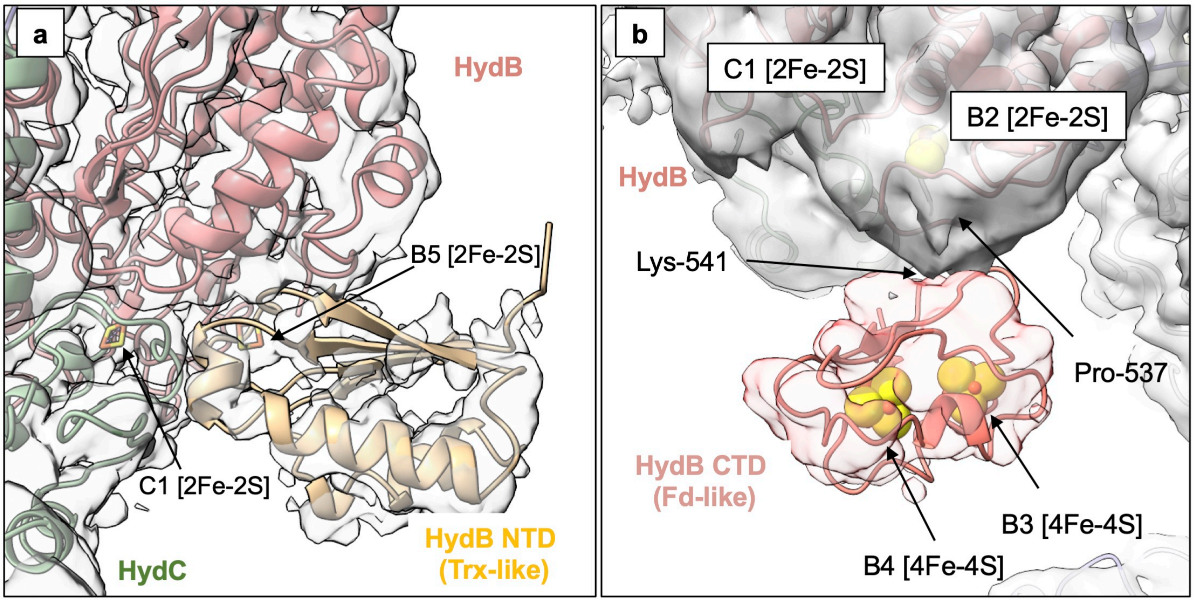

Supplementary Figure 9. Two local regions where the EM densities did not allow atomic-resolution structure model building in the HydB subunit. a) HydB NTD with a thioredoxin-like fold. The atomic model (yellow) is derived by comparative modeling in the Robetta, using the homolog domain of the *Rhodobacter capsulatus* formate dehydrogenase (PDB ID 6TGA) as a template. **b**) EM density of the HydB Fd-like CTD is shown in transparent salmon superimposed with the Robetta-derived model (salmon) using the homolog domain of the *Desulfovibrio vulgaris* electron transfer complex cytochrome C_3 and [Fe]-hydrogenase (PDB ID 1GX7). The B3 and B4 clusters in HydB CTD were placed based on their locations in the homolog structure. Pro537 is the last amino acid in the built model of HydB, the starting residue of the Fd-like CTD is Lys541.

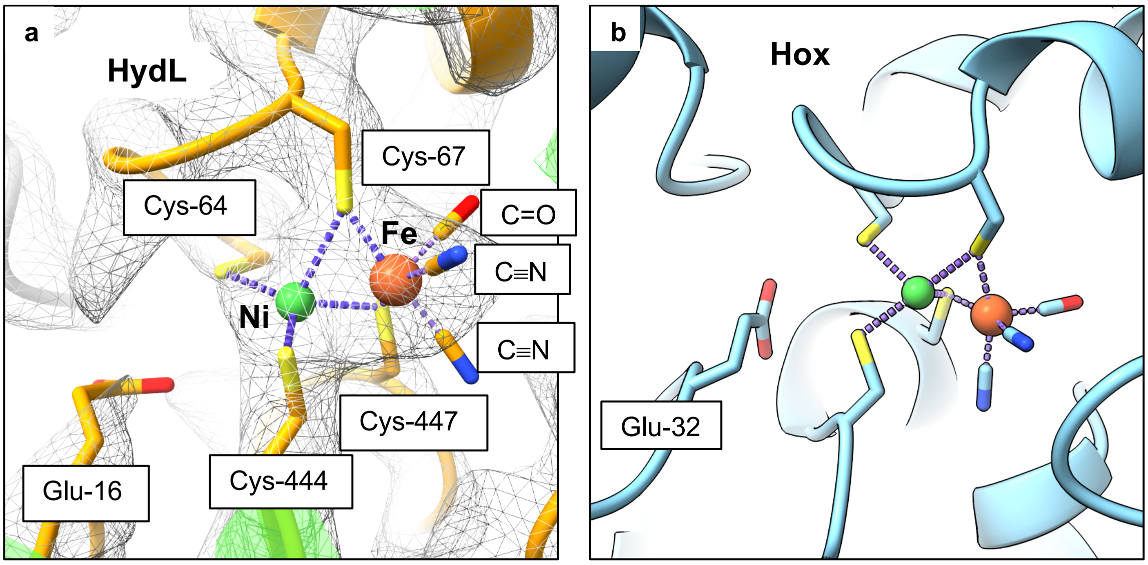

Supplementary Figure 10. Reduced state of the hydrogenase NiFe active site in HydL. The NiFe center in HydL stays in a reduced state (**a**) by comparing with the HoxH structure (PDB ID 5xfa) solved in the H2-reduced state (**b**). Based on the study of HoxH, the Glu16 will point to the nickel once the complex is air oxidized. The mesh in the panel (a) presents the electron density of the apo state.

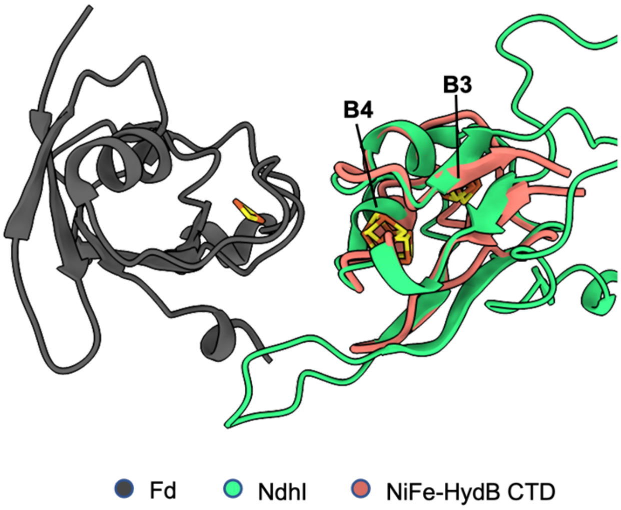

Supplementary Fig. 11. Potential binding site of ferredoxin (Fd) on CTD of NiFe-HydB. The putative binding mode of Fd:NiFe-HydB is based on the binding of Fd on NdhI subunit in the structure of Fd-dependent complex I (PDB ID 6KHI). We found that NiFe-HydB CTD aligns well with NdhI. The structural similarity suggests that HydB CTD with iron-sulfur clusters B3/B4 may interact with Fd in a similar manner.

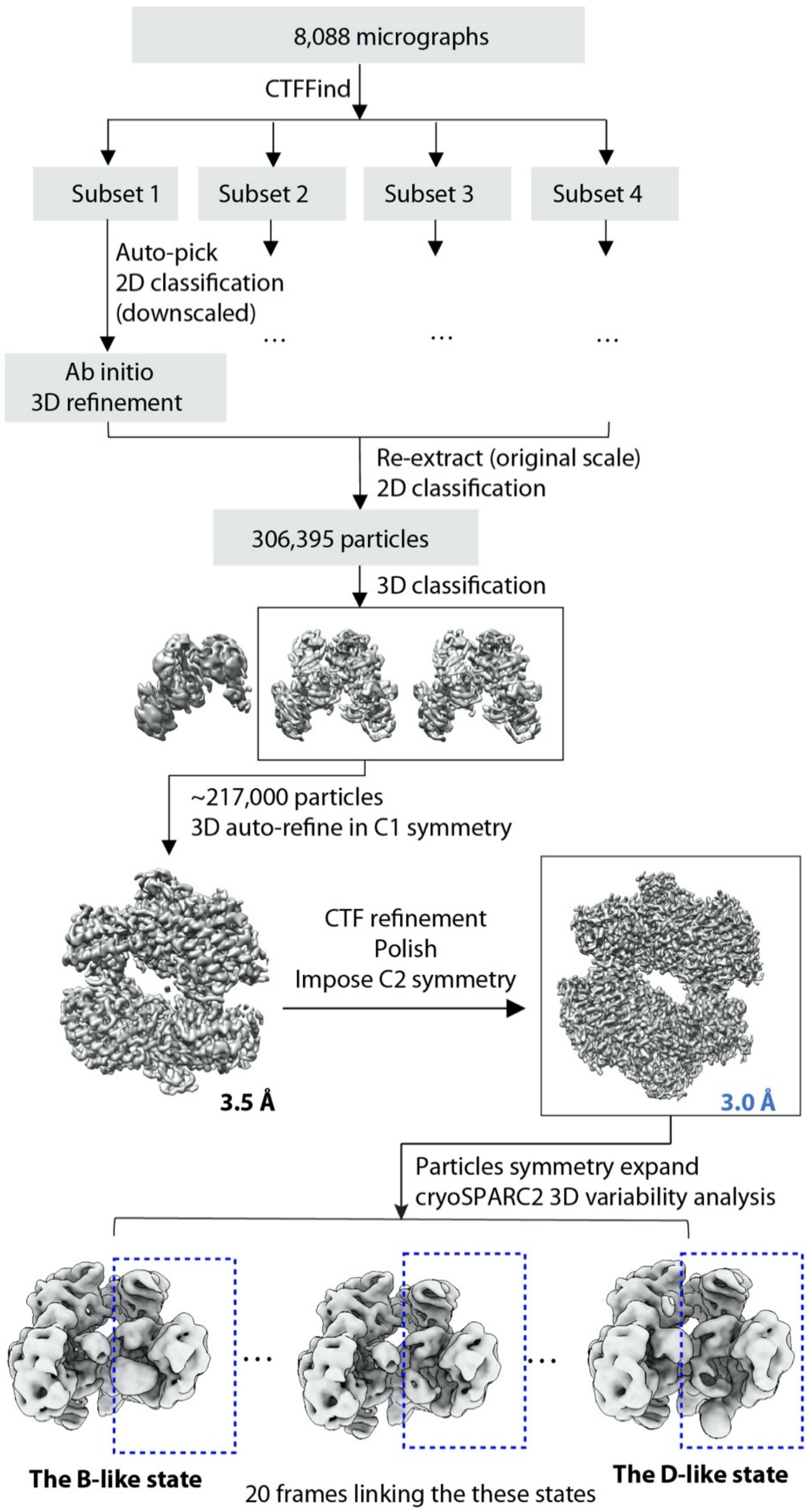

Supplementary Figure 12. Cryo-EM image processing and 3D reconstruction process of the NADH/FMN-bound NiFe-HydABCSL complex. We first refined the averaged structure at 3.0 Å resolution. Then 3D variability analysis was performed to the final dataset, leading to the identification of the B-like and D-like states. These states are linked to the

average state by a movie of two frames (3D maps). Note the 3D maps shown in three rows are in different views.

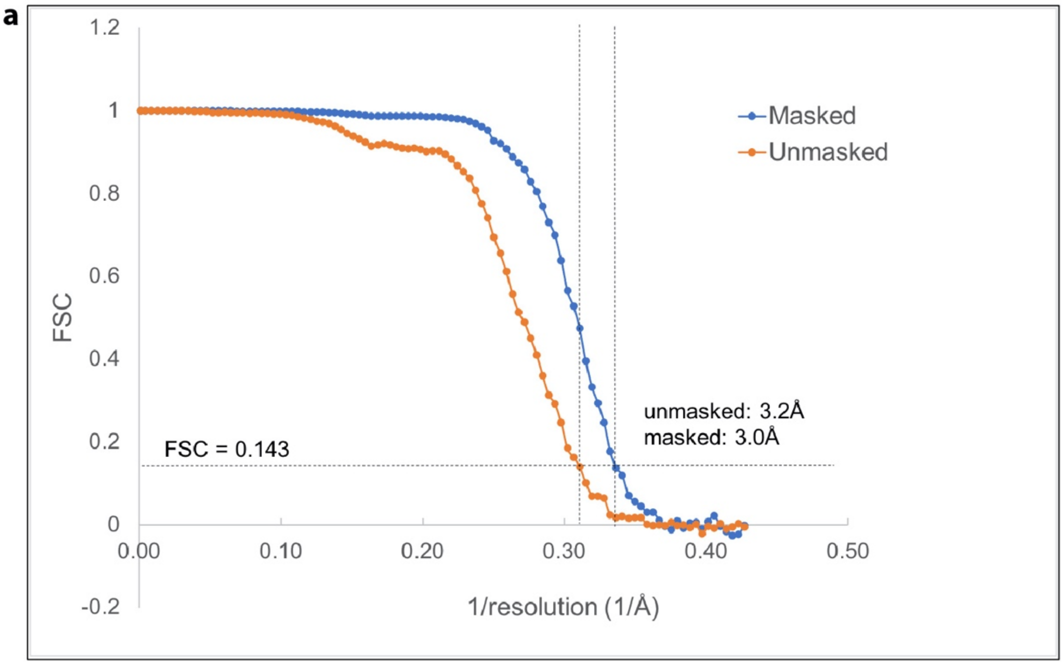

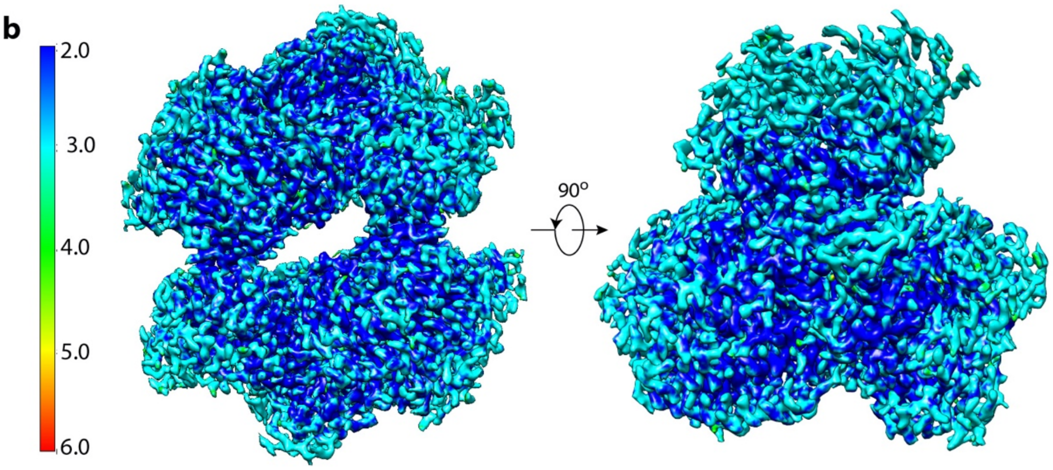

Supplementary Figure 13. Estimation of the resolution of the 3D map of the NADH/FMN-bound NiFe-HydABCSL. a) Gold-standard Fourier shell correlations of the masked and unmasked maps. **b**) Color-coded local resolution estimation.

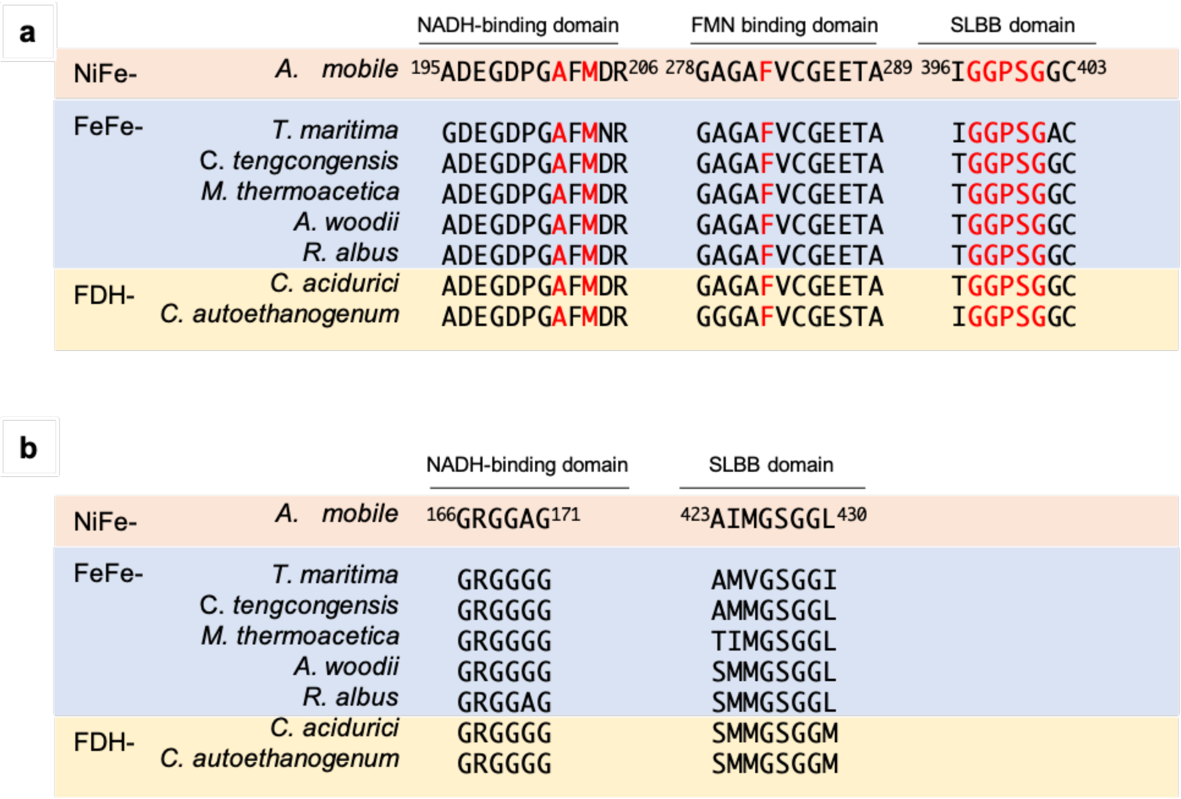

Supplementary Figure 14. Sequence alignment of the five NAD/FMN-binding loops in HydB-type subunits showing their conservation. a) Alignment of the three middle loops. The residues highlighted in red are conserved in bifurcating hydrogenase and are distinct from those in the non-bifurcating version based on the study of Losey et al including conserved AFM motif in NADH-binding domain, conserved phenylalanine in FMN binding domain and GGPSG motif in SLBB domain [1]. **b**) The first and the fifth loops in the binding site are also conserved.

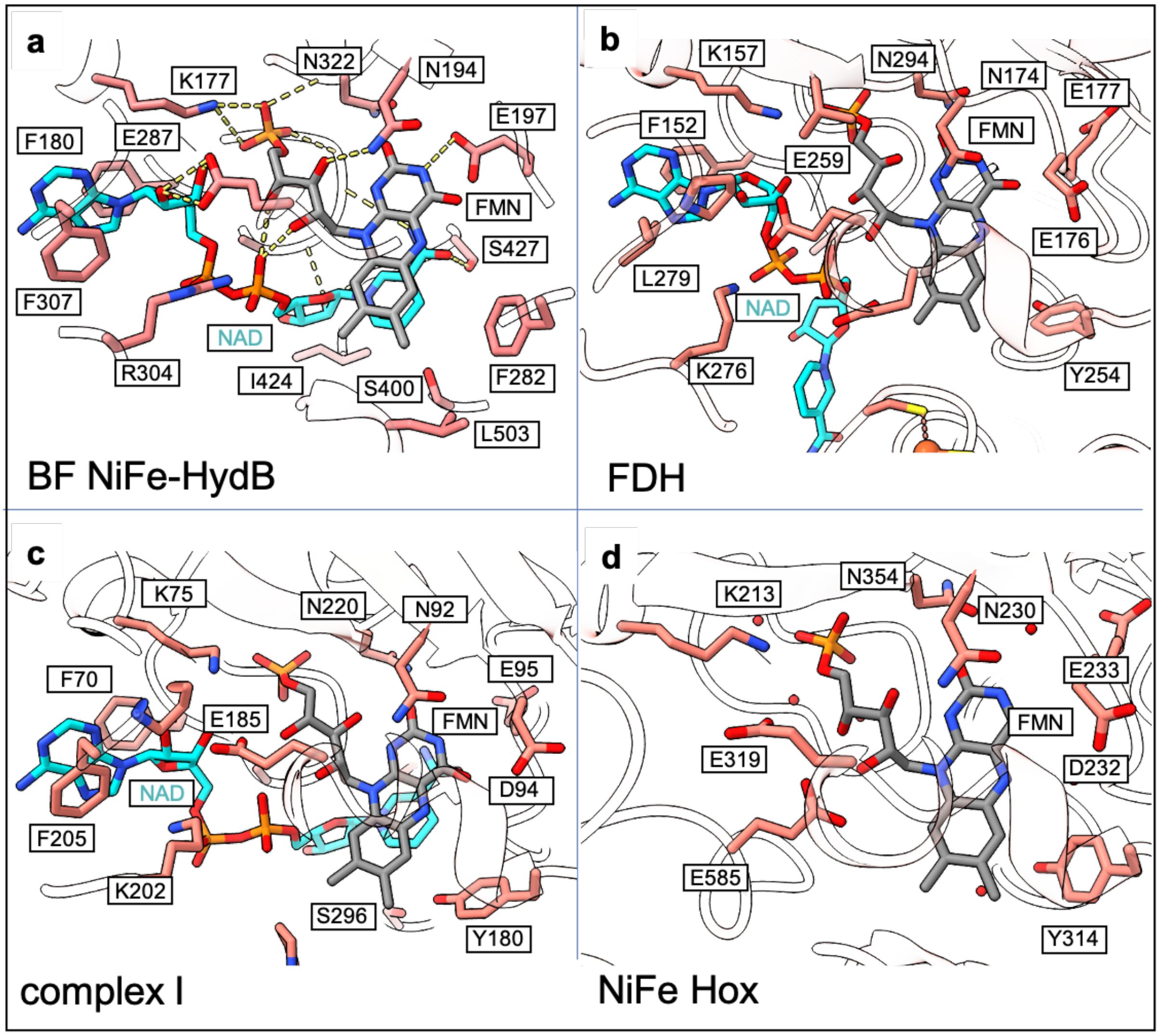

Supplementary Figure 15. Conserved FMN/NAD(H) binding pocket in homologue NAD-dependent oxidoreductase. The FMN/NAD(H) binding motifs in NiFe HydABCSL (**a**) are similar to those in the non-BF homologues: formate dehydrogenase (**b**, PDB ID 6TG9), the respiration complex I (**c**, PDB ID 3IAM), and the NiFe hydrogenase (**d**, PDB ID 5XF9). The reported NiFe Hox structure does not contain NAD(H). The Tyr residue at the lower right corner is universally conserved among all non-BF homologues (b-d); the corresponding residue is Phe-282 in the bifurcating NiFe-HydB (a).

Legend for Supplementary Video 1

Supplementary Video 1. 3D variability analysis reveals that the NAD/FMN-bound NiFe-HydABCSL fluctuates between a bifurcation-like and a transduction-like state. The video first shows the high-resolution 3D map rotating around a vertical axis, then switch to a lower resolution 3D variability analysis result, showing the transition from the BR-like to PB-like state, with changes primarily around the Fd-like CTDs of HydB and HydC.
